# Nanoplastic-induced microbiome shifts reduce *Daphnia* fitness and increase parasite reproduction

**DOI:** 10.1093/ismeco/ycag109

**Published:** 2026-04-20

**Authors:** Vanderville Villegas, Amruta Rajarajan, Erika Berenice Martínez-Ruiz, Kristel F Sánchez, Justyna Wolinska

**Affiliations:** Department of Evolutionary and Integrative Ecology, Leibniz Institute of Freshwater Ecology and Inland Fisheries (IGB), Berlin 12587, Germany; Institute of Biology, Freie Universität Berlin, Berlin 14195, Germany; Department of Evolutionary and Integrative Ecology, Leibniz Institute of Freshwater Ecology and Inland Fisheries (IGB), Berlin 12587, Germany; Department of Evolutionary and Integrative Ecology, Leibniz Institute of Freshwater Ecology and Inland Fisheries (IGB), Berlin 12587, Germany; Department of Evolutionary and Integrative Ecology, Leibniz Institute of Freshwater Ecology and Inland Fisheries (IGB), Berlin 12587, Germany; Department of Evolutionary and Integrative Ecology, Leibniz Institute of Freshwater Ecology and Inland Fisheries (IGB), Berlin 12587, Germany; Institute of Biology, Freie Universität Berlin, Berlin 14195, Germany

**Keywords:** host–microbiome, host–parasite, dysbiosis, 16S metabarcoding, microbiome transplant

## Abstract

Environmental pollutants can profoundly influence host-associated microbiomes, with cascading effects on host health and susceptibility to disease. Here, we investigated whether nanoplastic particles (NPs), a pervasive contaminant, influence host–parasite interaction by altering the microbiome of the water flea *Daphnia magna*. Microbiomes from NP-exposed and control *Daphnia* donors were transplanted to axenic *Daphnia* recipients, which were subsequently challenged with the fungal parasite *Australozyma monospora* sp. nov. Host and parasite fitness were then compared across treatments. NP exposure induced marked shifts in bacterial community structure and increased microbial diversity in donor microbiomes. These shifts persisted after transplantation, with recipient microbiomes remaining distinct from controls throughout the host lifespan, despite the absence of direct NP exposure. Microbiome shifts associated with NP exposure corresponded to elevated parasite reproduction and reduced host fecundity, while host survival was unaffected. Our findings demonstrate that NP pollution can indirectly compromise host health by reshaping microbial communities, highlighting microbiome-mediated pathways as important mechanisms through which emerging pollutants may shape ecological and evolutionary dynamics.

## Graphical Abstract

**Graphical Abstract:**
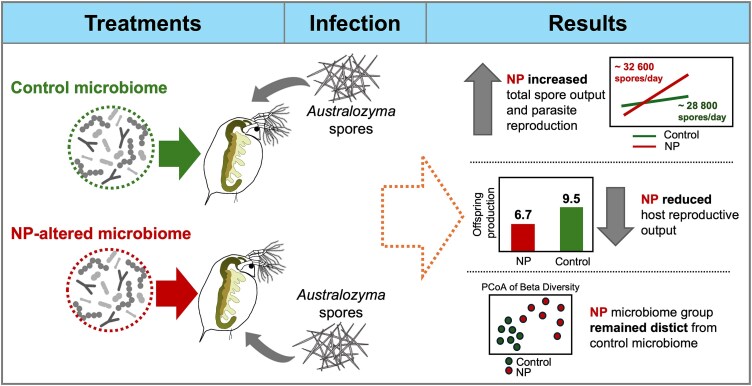

## Introduction

The Anthropocene is marked by the widespread accumulation of synthetic contaminants across ecosystems. Among these, plastic pollution has emerged as a pervasive threat to both terrestrial and aquatic environments [1, 2]. Nanoplastic particles (NPs), defined as plastic fragments ranging from 1 to 1000 nm in size [3], are of particular interest due to their ability to cross biological barriers [4]. They have been detected in remote environments [5] and across a wide range of organisms [6]. This widespread distribution reinforces global concern regarding the risks NPs pose to aquatic ecosystems and water quality [7].

Early research on NPs focused primarily on the toxicological effects at the level of individual species. However, attention has increasingly shifted towards their influence on species interactions, including interactions with host-associated microbiomes [8] and parasites [9]. The microbiome plays a central role in regulating host fitness [10], mediating immune defences against pathogens [11, 12], and facilitating environmental adaptation [12]. Disruptions in microbial community composition, referred to as ‘dysbiosis’, can compromise these functions and are suspected to increase host susceptibility to parasitic infections [13].

Despite growing recognition of microbiome importance, direct evidence connecting pollutant-induced microbial shifts to altered infection outcomes remains scarce. Host–parasite interactions are shaped by a complex interplay among host condition, parasite traits, and environmental context. The microbiome has recently been integrated into this framework as a central component of the ‘disease pyramid’ [14], emphasizing its integrative role in disease dynamics. In freshwater ecosystems, the well-established model involving *Daphnia magna* and the fungal parasite *Australozyma monospora* sp. nov. [15] (formerly *Metschnikowia bicuspidata*) offers a powerful system to investigate how environmental pollutants shape disease outcomes.

*Daphnia magna*, as a keystone species with critical ecological functions [16], serve as a sentinel organism for assessing microbiome-mediated effects of pollutants in freshwater ecosystems [17]. *Australozyma monospora* is a widespread and virulent parasite [18] that reduces host fitness [19], can alter host microbiome composition [20], and exhibits infection severity that is sensitive to environmental conditions [21]. Transmission occurs via spores released from decomposing hosts [22]; following ingestion, spores mechanically breach the gut epithelium and proliferate within the host haemolymph [23]. This well-characterized system provides a tractable model to explore how pollutant-driven microbiome shifts affect host fitness and disease dynamics, with broader ecological and evolutionary consequences.

Exposure to NPs has been shown to alter the *Daphnia* microbiome [8] and to increase susceptibility to *Australozyma* infections [8, 9, 24]. However, the casual role of NP-induced microbiome alterations in mediating infection outcomes remain unexplored. This gap limits our understanding of how emerging pollutants modulate host–parasite interactions through indirect microbiome-mediated alteration.

Here, we directly tested whether NP-induced microbiome alterations are responsible for increased infection susceptibility and altered host fitness. Using a microbiome transplantation approach, we disentangled the effects of NP-altered microbial communities from direct NP exposure. Microbiomes from NP-exposed and unexposed control donor *D. magna* were transferred to axenic recipients, which were subsequently challenged with *A. monospora* spores. We assessed whether recipients of NP-altered microbiomes differed from the recipients of control microbiomes in microbial diversity and composition, and whether these differences translated into changes in parasite infection intensity and host fitness. Specifically, we hypothesized that (i) recipients of NP-altered microbiomes would exhibit increased susceptibility to *Australozyma* infection and enhanced parasite proliferation, (ii) these recipients would have reduced fecundity and survival, and that (iii) NP-altered microbiomes would remain structurally distinct and exhibit greater temporal instability throughout the host lifespan compared to controls. Together, these hypotheses position the microbiome as a central mediator of pollutant effects on host–parasite interactions and highlight its potential role in amplifying the ecological consequences of nanoplastic pollution.

## Materials and methods

### Study organisms

We used *D. magna* genotype E17:07, originally isolated from a temporary pond near Oxford, England. This genotype is highly susceptible to infection by the fungal parasite *A. monospora* sp. nov. and has been previously used in studies on *Daphnia*–*Australozyma*–NP interactions [8, 9, 24]. Cultures were maintained in glass jars containing 250 ml of synthetic *Daphnia* medium (SSS medium) [25], under a 12:12 light–dark cycle, and fed daily with green algae *Tetradesmus obliquus* at 1 mg C l^−1^.

The *Australozyma* strain ‘VRANOV_2018’ was originally isolated from the Vranov Reservoir, Czech Republic, in 2018 and subsequently maintained *in vivo* on *D. magna* E17:07 [26]. *Australozyma* is a horizontally transmitted, lethal parasite infecting various *Daphnia* species in lakes and ponds [18, 19]. Visible infection typically occurs 9–10 days post-exposure and is characterized by opaque regions in the host body cavity caused by dense spore aggregation, readily detectable under a stereomicroscope [23].

### NP suspension

We used spherical polystyrene nanoparticle beads (Micromod Partikeltechnologie GmbH, Germany, product name: micromer-greenF, product code: 29-00-501) with a nominal diameter of 50 nm. Detailed particle characteristics are provided in [8]. This NP size (50 nm) and concentration (5 mg l^−1^) were selected as they induce pronounced microbiome shifts in *D. magna*, compared to lower concentrations (1 mg l^−1^) or larger particles (100 nm) [8], and have previously been shown to affect infection outcomes in the *Daphnia–Australozyma* system [9, 24, 27]. Although this concentration exceeds most reported environmental levels (~1.5 mg l^−1^ total polymers [28]), nanoplastics remain difficult to detect and quantify in natural systems [29], and locally elevated concentrations may occur near pollution hotspots. Thus, applied concentration represent a worst-case exposure scenario. A working suspension of 5 mg l^−1^ in SSS medium was prepared from stock solution 24 h prior to use and stored at 19°C in darkness to allow equilibration.

### *Daphnia* microbiome donors

Second-clutch female juveniles (<24 h old) from synchronized mothers were assigned to two treatment groups with 32 replicates each: (i) control microbiome donors and (ii) NP-altered microbiome donors exposed to 5 mg l^−1^ 50 nm NPs for 16 days [8]. Individuals were maintained separately in glass tubes containing 15 ml SSS medium and fed daily with *T. obliquus* (1 mg C l^−1^). Medium (control and NP treatments) was refreshed every 4 days due to NP aggregation with algal biomass. All cultures were maintained at 19°C under a 12:12 light–dark cycle.

### *Daphnia* axenic recipients

Axenic recipients were generated from second-clutch eggs collected 12–23 h after deposition. Eggs were isolated under a stereomicroscope using tweezers and a glass micropipette, rinsed in medium, and transferred to sterile six-well plates (30 eggs per well; 6 ml fresh SSS). All subsequent procedures were conducted in a biosafety cabinet. Eggs were surface sterilized using a 0.25% glutaraldehyde for 10 min based on previous studies [30, 31] and preliminary experiments, followed by two 10-min rinses in autoclaved SSS medium. This approach ensured effective bacterial removal while maintaining high egg viability (~90%). Sterilized eggs were placed in fresh, autoclaved SSS medium in sterile 6-well plates, sealed with parafilm, and incubated at 19°C under a 12:12 h photoperiod until hatching.

### Verification of axenicity

Axenicity was verified by inoculating eggs and media (axenic and non-axenic controls) into LB broth (six replicates each, with five eggs per replicate). Optical density (OD_600_) was measured weekly for 4 weeks (spectrophotometer HACH LANGE GmbH, Berlin, Germany). Bacterial growth occurred only in LB cultures inoculated with non-axenic eggs or non-autoclaved medium, while no growth was detected in axenic eggs or autoclaved medium (Fig. S1A and B), confirming that the axenization protocol was effective.

### Microbiome transplant procedure

Donor *Daphnia* from each treatment were rinsed in sterile SSS medium and pooled into 2-ml reaction tubes. All donors were 16-day-old adults. This age was selected because previous work demonstrated that exposure to 50 nm NPs (5 mg l^−1^) for 16 days induces significant alterations in the *Daphnia* microbiome [8]. For microbiome transplantation, donors were pooled at a fixed donor-to-recipient ratio (one adult donor per three recipient juveniles): 10 control donors were used to inoculate 30 axenic juveniles, and 24 NP-exposed donors were used to inoculate 72 axenic juveniles. Donors were homogenized using a sterile pestle. To remove residual NP particles from the NP-exposed microbiomes and co-transfer of dissolved metabolites, homogenates were passed through sterile 0.2-μm polycarbonate filters and washed with 500 μl of sterile SSS medium (a similar procedure was applied to control microbiomes). Microbial communities were then backwashed from the filters in two 500-μl steps (final volume: 1 ml) and transferred into new sterile 2-ml tubes.

Axenic juveniles were individually transferred to glass vials containing 5 ml sterile SSS medium in glass vials and inoculated with the respective microbiome suspension. Recipients were incubated with the transplanted microbiomes for 48 h: unfed on day 1, then fed with autoclaved *T. obliquus* (2 mg C l^−1^) on day 2. The increased food concentration compensated for potential quality loss due to autoclaving. The success of microbiome transplantation in both treatments was verified by 16S metabarcoding (see following section). The control microbiome treatment included 20 replicates, whereas the NP-altered microbiome treatment included 51 replicates, reflecting an *a priori* adjustment to account for expected higher mortality in this group.

### Microbiome-recipient experiment

**Figure 1:**
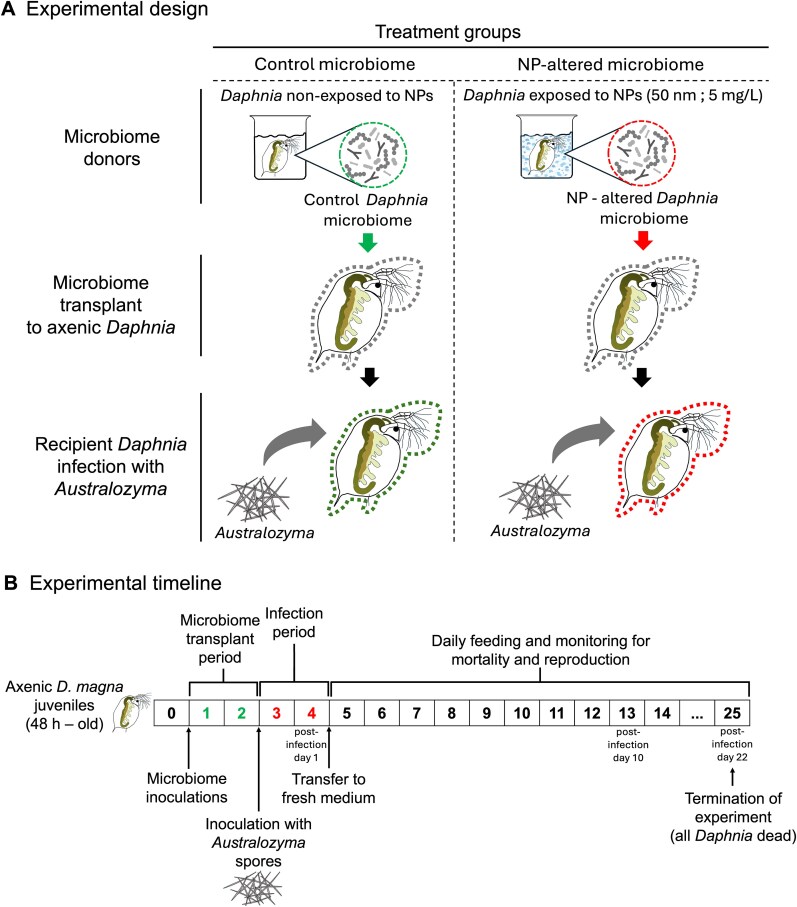
Schematic overview of (A) experimental design and (B) experimental timeline. Two treatments were used: ‘Control microbiome’ (axenic *Daphnia magna* receiving microbiomes from unexposed donors) and ‘NP-altered microbiome’ (axenic *Daphnia* receiving microbiomes from NP-exposed donors), followed by exposure to the fungal parasite *Australozyma monospora*. The timeline shows key steps: microbiome transplantation, parasite inoculation, and daily monitoring of survival and reproduction. The experiment concluded on day 25 (22 days post-infection), when all individuals had died.

Following the 48-h inoculation period, recipients were exposed to mature *A. monospora* spores (Fig. 1)*.* Spores were obtained from infected *D. magna* from stock cultures, homogenized using a pestle in a 2-ml tube with defined volume, filtered through a 0.7-μm cellulose nitrate filter to remove bacteria, washed twice with sterile SSS medium, and resuspended in 1.5 ml SSS medium. Spore concentration was quantified using an improved Neubauer chamber under 100× phase contrast (Carl Zeiss Axioscope, Gottingen, Germany). Each animal was exposed to ~3500 spores ml^−1^ for 48 h in 5 ml SSS medium. This dose has been shown to reliably generate high infection prevalence [8, 24]. Animals were unfed on the first exposure day and fed with autoclaved *T. obliquus* (2 mg C l^−1^) on the second day. Post-exposure, individuals were transferred to sterile 250-ml screw-cap glass bottles containing 50 ml SSS medium and fed daily (2 mg C l^−1^ autoclaved algae). Survival was monitored daily, and offspring were counted and removed. Upon death, individuals were examined for infection under a stereomicroscope. Infected hosts were homogenized; 100-μl subsamples were fixed in 5% formaldehyde for spore quantification, and the remaining material was stored at −20°C for microbiome analysis. The experiment ended on day 25 (day 22 post-infection), when all animals had died (Fig. 1).

### Parasite fitness

Individuals that died before day 10 post-infection without visible signs were excluded, as infection cannot be reliable confirmed earlier. All individuals surviving beyond day 10 were infected. *Total spore output* per infected host was determined by counting mature spores (samples processed blindly). Normality (Shapiro–Wilk) and variance homogeneity (Levene’s test) were assessed. Group differences were tested using Welch’s two-sample *t*-test (R car package [32]). To evaluate whether the relationship between host lifespan post-infection and spore output differed between treatments, a linear regression model including a treatment × post-infection lifespan interaction was applied.

### Host fitness

*Host fecundity* was quantified as total offspring produced per individual. Individuals that died before infection but were old enough to reproduce (≥8 days) were retained. Data normality and homogeneity were assessed as above, and differences were tested using a two-sample *t*-test. Survival (days post-infection) was analysed using Kaplan–Meier curves (R survival package [33]) and an accelerated failure time (AFT) model, using the survival [33] and flexsurv [34] R packages. The AFT model was selected to estimate variations in survival duration rather than hazard rates, providing a more intuitive understanding of treatment effects on lifespan [35]. All analyses were conducted in R v4.3.1 [36] within RStudio v.2024.12.0 + 467 [37].

### Sample sequencing for bacterial community profiling

To assess NP-induced microbiome alterations and verify microbiome establishment in recipients, six donor individuals per treatment (NP-exposed and controls) and four recipient individuals per treatment (48 h post-inoculation) were sampled. Infected recipient samples from the treatment groups (control microbiome: 20, NP-altered: 47) were also collected for microbiome analyses.

DNA was extracted using the DNeasy PowerSoil Pro Kit (Qiagen Cat. No. 47106). DNA concentrations were quantified using QuantiFluor dsDNA dye (Promega, USA) and Quantus Fluorometer (Promega, USA). The V4–V5 region of the 16S rRNA gene was amplified using primers 515F (5′-GTGCCAGCMGCCGCGGTAA-3′) [38] and m806R (5′-GGACTACNVGGGTWTCTAAT-3′) [39]. The PCR protocol included an initial denaturation step at 98°C for 2 min, followed by 30 cycles of denaturation at 98°C for 10 s, annealing at 57°C for 20 s, and elongation at 72°C for 20 s, with a final elongation at 72°C for 2 min. Triplicate PCRs per sample were pooled and purified [40]. Each 25-μl PCR mix consisted of 6.75 ng DNA, 5 μl of Q5 reaction buffer (New England Biolabs, Ipswich, MA, USA), 1.25 μl (10 μM) of each primer (biomers.net, Ulm, Germany), 0.5 μl (10 mM) of a deoxynucleotide (dNTP) solution mix (New England Biolabs), 0.25 μl (2 U/μl) of Q5 Hotstart High-Fidelity DNA Polymerase (New England Biolabs), and nuclease-free water. The PCR products were purified using CleanPCR magnetic beads (CleanNA, Waddinxveen, The Netherlands), quantified, and stored at −20°C. Eight negative controls were included: four extraction blanks and four no-template PCR controls.

Indexing followed the Illumina 16S library preparation protocol [41], with Q5 High-Fidelity polymerase (New England Biolabs). Each indexing 25-μl PCR mix consisted of 10 μl (10 ng/μl) template (purified target PCR products), 5 μl of Q5 reaction buffer (New England Biolabs), 1.25 μl (10 μM) of each Nextera Index Kit primers P5 and P7 (Illumina Inc., San Diego, CA, USA), 0.5 μl (10 mM) of a dNTP solution mix (New England Biolabs), 0.25 μl (2 U/μl) of Q5 Hotstart High-Fidelity DNA Polymerase (New England Biolabs), and nuclease-free water. Indexed PCR products were purified twice using the CleanNGS magnetic beads (GC Biotech, Waddnixveen, The Netherlands), quantified, and normalized to form an equimolar pool. The final pool was purified once more before sequencing. Sequencing was performed at the Berlin Center for Genomics in Biodiversity Research (BeGenDiv, Berlin, Germany) on the Illumina MiSeq platform (v3 600 cycles).

### Pre-processing of reads

Reads were processed using DADA2 [42] in R v4.3.1 within RStudio v.2024.12.0 + 467. Quality assessment was performed on forward and reverse reads for each sequencing run, followed by primer trimming (first 20 bases) and truncation (270 bp forward; 220 bp reverse), dereplication, and merging. Sequence tables from two runs were combined, and chimeras were removed using a *de novo* approach. Taxonomic assignment of amplicon sequence variants (ASVs) was conducted using the SILVA prokaryotic SSU taxonomic training dataset v138.2 [43], formatted for the DADA2 pipeline [42], using the *assignTaxonomy* function. Downstream analyses were performed using the phyloseq R package [44]. ASVs unclassified at the phylum level (*n* = 125) or identified as chloroplasts (*n* = 14) or mitochondria (*n* = 46) were removed. Samples were rarefied using the *rarefy_even_depth* function from the vegan package [45], resulting in 545 ASVs.

### Microbiome data analyses

Analyses were conducted in R v4.3.1 within RStudio v.2024.12.0 + 467. Data visualizations was performed using the ggplot2 package [46]. Negative controls differed markedly from biological samples (Fig. S2) and were therefore excluded from downstream analyses.

*Alpha diversity*: ASV richness and Inverse Simpson Index (reflecting evenness) were computed using the *estimate_richness* function in the phyloseq package. Generalized linear models with appropriate error distributions (based on dispersion diagnostics) were used to test the effects of treatment, days lived post-infection, and their interaction.

*Beta diversity*: Jaccard and Bray–Curtis distances were calculated with *phyloseq::distance*. Community differences were analysed using PERMANOVA (*adonis2* function, vegan package, 9999 permutations), with terms evaluated sequentially and marginal effects tested. Distance-based redundancy analysis (db-RDA) was performed using the *capscale* function from the vegan package to assess constrained variation, with significance tested via ANOVA-like permutation tests (9999 permutations). Community structure was visualized using principal coordinates analysis (PCoA). Associations between community structure and host lifespan post-infection as well as parasite spore output (proxy for parasite fitness) were examined by fitting environmental vectors using *envfit* function from the vegan package (999 permutations). Significant correlations were visualized as directional arrows in ordination plots.

*Indicator taxa*: Indicator species analysis was conducted using *signassoc* function of indicspecies package [47] with 9999 permutations and Sidák correction for multiple testing. Heatmaps were generated to visualize relative abundances of ASVs significantly associated with either treatment. Each ASV identified as indicator taxa—either uniquely present or absent in the NP-altered group compared to the control—was BLASTed against the NCBI database to determine their closest taxonomic matches.

*Differential abundances of bacterial orders*: ASVs were aggregated by the order level. Low-abundance taxa (<1% total reads) and taxa not present across all samples were grouped as ‘Other’. Differential abundance analyses were conducted using the Wald test implemented in the DESeq2 package [48], with the ‘poscounts’ method for size factor estimation and false discovery rate correction for multiple comparisons. Analyses were conducted (i) across all samples for overall treatment comparisons and (ii) separately for days 15, 17, and 20 post-infection. For time-specific analyses, treatment and days lived post-infection were combined into a single factor, followed by pairwise comparisons. Compositional means and standard deviations of bacterial orders were calculated for the full dataset and separately for days 15, 17, and 20 post-infection using the microbiome package [49].

### Functional prediction and differential abundance analysis from 16S rRNA gene data

Predicted functional profiles for KEGG Orthologs (KOs) and Enzyme Commission numbers (ECs) were inferred from 16S rRNA gene sequences using PICRUSt2 v2.6.3 [50]. KO and EC abundance tables were imported into R for downstream analyses. KO abundances were log2-transformed and analysed using limma [51] to model continuous abundance data, while EC counts were analysed using DESeq2 [48]. Differential features were identified at an adjusted *P*-value < .05, with directionality (enriched in NP or in control) determined based on fold changes. Significant KOs and ECs were annotated with KEGG descriptions and mapped to pathways using the KEGGREST package [52] via *keggList* and *keggGet* functions. Sample clustering and ordination were assessed with PCA using *prcomp* function, and differential abundance results were visualized with volcano plots using ggplot2 [46].

## Results

### Parasite fitness increases in recipients of NP-altered microbiomes

**Figure 2:**
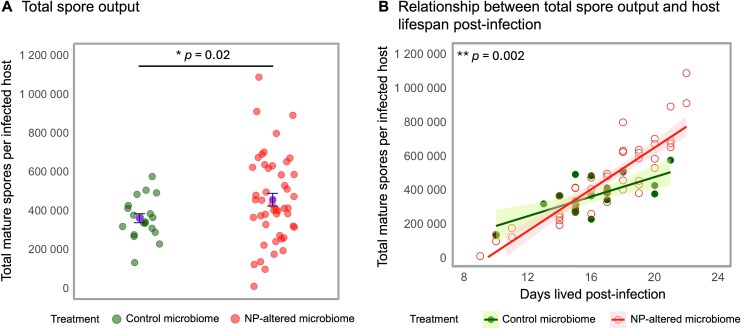
Effect of the microbiome transplant on parasite infection in recipient *Daphnia*, depending on whether donor microbiomes were NP-exposed or unexposed (see Fig. 1). (A) Total parasite spore output per host, measured as the number of mature transmission spores at death. (B) Relationship between spore output and host lifespan post-infection. Each point represents an individual microbiome recipient. Treatment means ± standard errors are shown. In panel B, solid circles represent the control group, and open circles represent the NP-altered group. Solid lines show regression fits for both groups. Shaded areas denote 95% confidence intervals for each regression.

*Daphnia magna* hosts that received NP-altered microbiomes produced 27% more *A. monospora* spores than those receiving control microbiomes (Fig. 2A, Table 1A). Linear regression of spore load against host lifespan post-infection revealed distinct infection dynamics between the two groups (Fig. 2B). In both groups, spore load increased significantly with host lifespan; however, the slope was steeper in recipients of the NP-altered microbiomes (~32 600 spores/day) compared to controls (~28 800 spores/day) (Fig. 2B, Table 1B). This pattern indicates enhanced parasite proliferation in recipients of NP-altered microbiomes.

**Table 1:** Statistical results comparing parasite fitness between control and NP-altered microbiome-recipient treatments: (A) Welch’s *t*-test for total spore output per infected host and (B) linear regression testing the relationship between total spore output and days lived post-infection.

(A) Total spore output per infected host (Welch’s *t*-test)							
Treatment	*n*	Mean	*df*	*t*-value	95% confidence interval		*P*-value
					Upper	Lower	
Control microbiome	20	358 480.7	64.471	−2.381	−15 399.2	−175 765.9	**.020**
NP-altered microbiome	47	454 063.3					
**(B) Linear regression analysis results for the relationship between total spore output and host survival post-infection**							
				**Estimate**	**Std. error**	***t*-value**	***P*-value** **Pr(>|*t*|)**
(Intercept)				−102 408	145 268	−0.705	.483
**Treatment**				−478 416	164 372	−2.911	**.005**
**Days lived post-infection**				28 806	8975	3.210	**.002**
**Treatment × days lived post-infection**				32 608	10 030	3.251	**.002**

‘Treatment’ compares the ‘NP-altered microbiome’ group against the reference ‘Control microbiome’ group. The interaction term (Days lived post-infection × Treatment) indicates whether the effect of host lifespan on spore output differs between treatments. Significant effects [*P*-value, Pr(>|*t*|) < .05] are shown in bold. Model statistics: adjusted *R*^2^ = 0.7597, *F*-statistic = 70.54 on *df* = 3 and 63, ***P* < 2.2e−16**.

### Recipients of NP-altered microbiomes produce fewer offspring

**Figure 3:**
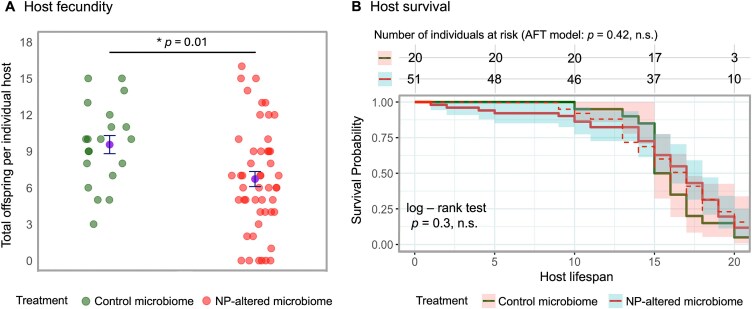
Effect of microbiome transplant on host fitness, depending on whether donor microbiomes were NP-exposed or unexposed (see Fig. 1). (A) Host fecundity, measured as the total number of live offspring produced. Each point represent an individual microbiome recipient and are grouped as in Fig. 2; treatment means ± standard errors are shown. (B) Host survival post-infection, measured as the number of days each host lived after parasite exposure. Survival curves were generated using the Kaplan–Meier estimator and modelled with an accelerated failure time (AFT) model. Shaded areas indicate 95% confidence intervals. Number of individuals at risk are shown above the plot.

In addition to higher infection burdens, recipients of NP-altered microbiomes showed reduced reproductive output, producing on average 6.7 offspring versus 9.5 in the control group (Fig. 3A, Table 2A). Host survival did not differ significantly between treatments (Fig. 3B, Table 2B), indicating that reduced fecundity was not attributed to differential mortality.

**Table 2:** Statistical results comparing host fitness between control and NP-altered microbiome-recipient treatments: (A) two-sample *t*-test for total offspring production and (B) log-rank test and accelerated failure time (AFT) model for differences between treatments.

(A) Total offspring (two-sample *t*-test)							
Treatment	*n*	Mean	*df*	*t*-value	95% confidence interval		*P*-value
					**Upper**	**Lower**	
Control microbiome	20	9.6	67	2.622	4.994	0.677	**.011**
NP-altered microbiome	49	6.7					
**(B) Host survival [log-rank test and AFT model]**							
**Log-rank test**							
**Treatment**		***N***	**(*O* − *E*)^2^/*E***	**(*O* − *E*)^2^/*V***	**Chi-sq (*df*)**	***P*-value**	
Control microbiome		20	0.770	1.3	1.3 (1)	.3	
NP-altered microbiome		51	0.232	1.3			
**AFT model**							
		**Estimate**	**Std. error**	***z*-value**	**Chi-sq (*df*)**	***P*-value**	
Treatment (Control vs. NP-altered)		0.047	0.059	0.8	0.62 (1)	.42	
Model summary: Log-likelihood					−206 (1)		

Significant effects (*P*-value <.05) are shown in bold.

### Recipient microbiomes differ in diversity and composition depending on donor NP exposure

Sequencing across two runs yielded ~5.9 million reads. Biological samples ranged from 29 395 to 158 840 reads, whereas negative controls yielded 476–3490 reads. NP exposure significantly altered donor microbiomes (Fig. S3A–D; Tables S1A and B; S2A and B), consistent with previously reported NP-induced shifts in the same *D. magna* genotype exposed to 5 mg l^−1^ of 50 nm NPs [8]. In recipients, neither ASV richness nor Inverse Simpson Index differed significantly between NP-altered and control microbiome groups after the 48-h inoculation period (Fig. S3A and B; Table S1A and B). However, beta diversity showed clear differences between recipient groups (Fig. S3C and D; Table S2A and B), confirming successful establishment of distinct microbiomes reflective of their respective donors.

**Figure 4:**
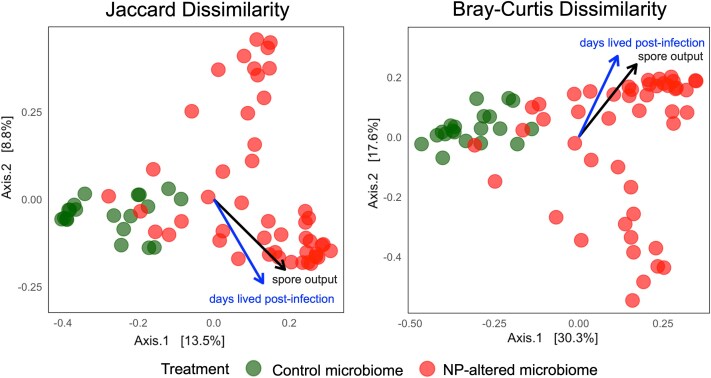
Principal coordinates analysis (PCoA) showing differences in microbiome composition between recipient *Daphnia* receiving NP-altered or control microbiomes (see Fig. 1). Plots are based on Jaccard dissimilarity (left) and Bray–Curtis dissimilarity (right). Each point represents an individual microbiome recipient from either the NP-altered or control group. Environmental vectors indicate variables correlated with microbiome variation. Percentages of axes indicate variance explained.

At the time of host death, beta-diversity patterns continued to show separation between treatments (Fig. 4). PCoA showed clear clustering along axis 1, explaining 13.5% (Jaccard) and 30.3% (Bray–Curtis) of the variation. Environmental vector fittings indicated that both spore output and days lived post-infection were associated with microbiome structure. PERMANOVA and db-RDA confirmed significant effects of microbiome treatment and host lifespan on community composition (Table 3A and B). Alpha diversity changed over time: recipients of NP-altered microbiomes initially exhibited lower ASV richness and evenness; however, both metrics increased more steeply over time and eventually exceeded those of control recipients (Fig. S4, Table S3A and B).

**Table 3:** PERMANOVAs and db-RDA results for beta-diversity metrics in *Daphnia* microbiomes between control and NP-altered recipient treatments: (A) Jaccard dissimilarity and (B) Bray–Curtis dissimilarity.

(A) Jaccard dissimilarity					
PERMANOVA					
	*df*	SumOfSqs	*R* ^2^	*F*	Pr(>*F*)
**Model**	3	3.855	0.158	3.930	**1e−04**
Residual	63	20.597	0.842		
Total	66	24.452	1.000		
**db-RDA**					
		***df***	**SumOFSqs**	***F***	**Pr(>*F*)**
**Treatment**		1	2.452	7.500	**.0001**
**Days lived post-infection**		1	0.976	2.985	**.0001**
Treatment × days lived post-infection		1	0.426	1.304	.0970
Residual		63	20.597		
**(B) Bray–Curtis dissimilarity**					
**PERMANOVA**					
	***df***	**SumOfSqs**	***R* ^2^**	***F***	**Pr(>*F*)**
**Model**	3	4.263	0.311	9.465	**1e−04**
Residual	63	9.459	0.689		
Total	66	13.722	1.000		
**db-RDA**					
		***df***	**SumOFSqs**	***F***	**Pr(>*F*)**
**Treatment**		1	2.983	17.315	**.0001**
**Days lived post-infection**		1	1.060	6.154	**.0001**
Treatment × days lived post-infection		1	0.256	1.488	.1349
Residual		63	10.852		

Analyses were performed with 9999 permutations. Significant effects (*P*-value: Pr(>*F*) < .05) are shown in bold.

### Recipients of NP-altered microbiomes exhibit distinct indicator taxa and differential abundance patterns

**Figure 5:**
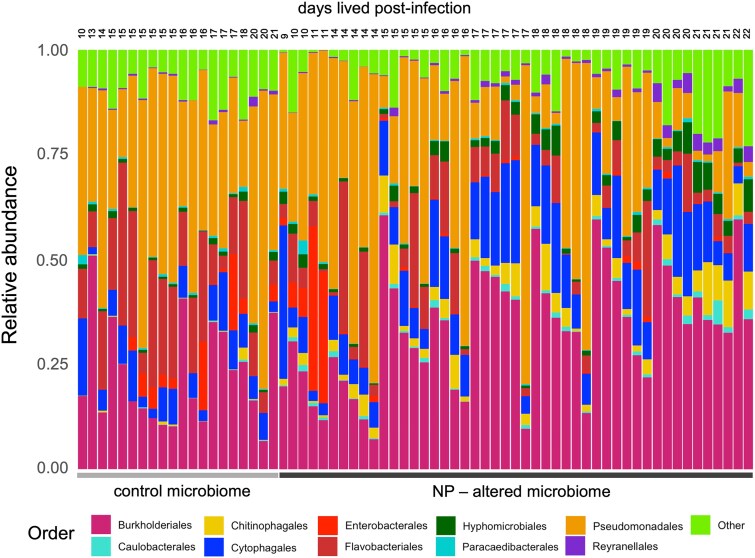
Relative abundance of bacterial orders in individual recipient samples across treatment groups. The *y*-axis represents relative abundance, and the *x*-axis groups samples by treatment. The top axis shows the number of days each host survived post-infection, ordered from earliest to latest death. Bacterial orders contributing <1% of total abundance and those not consistently present across all samples are grouped as ‘other’. Microbiomes were dominated by Burkholderiales, Caulobacterales, Chitinophagales, Cytophagales, Enterobacterales, Flavobacteriales, Hyphomicrobiales, Paracaedibacterales, Pseudomonadales*,* and Reyranellales, in addition to rare taxa (others), each represented by distinct colours.

Indicator species analysis identified specific ASVs associated with each treatment (Fig. S5, Table S4). Recipients of NP-altered microbiomes harboured 16 ASVs uniquely present and lacked 8 ASVs present in controls (closest identities and ecological functions summarized in Table S5). Additionally, 32 ASVs showed increased abundance and 15 showed reduced abundance in the NP-altered group (Fig. S5). Differential abundance analysis at the order level revealed that Chitinophagales, Caulobacterales, Hyphomicrobiales, and Reyranellales were significantly enriched in recipients of NP-altered microbiomes, while Flavobacteriales, Enterobacterales, and Paracaedibacterales were more abundant in control recipients (Fig. 5, Table S6A). These patterns were consistent across multiple time points. In particular, Chitinophagales and Caulobacterales remained enriched in the NP-altered group at days 15, 17, and 20 post-infection (Table S6B–D). In contrast, Flavobacteriales and Enterobacterales declined over time in this group.

### Functional predictions reveal broad microbiome remodelling in recipients of NP-altered microbiomes

Predicted functional profiles inferred from 16S rRNA sequences reveal widespread shifts in microbiome functional potential associated with NP exposure. A total of 2574 features were significantly different at the KO level (adjusted *P* < .05), with 1627 enriched in recipients of NP-altered microbiome and 947 enriched in control recipients (Fig. S6A, Supplementary Information 1). PCA of log2-transformed KO abundances showed clear separation between groups (PC1 = 23.7%, PC2 = 22.1%) (Fig. S6C), confirming NP-driven functional shifts. Similarly, 1141 ECs were significantly altered, with 761 enriched in NP-altered microbiomes and 380 enriched in controls. PCA of variance-stabilized EC abundances also separated treatments (PC1 = 30.8%, PC2 = 14.4%) (Fig. S6B and D, Supplementary Information 1).

Across both KO and EC annotations, significant features were predominantly assigned to broad metabolic categories, with *metabolic pathways* comprising the largest proportion in both treatments (KOs: ~20%; ECs: 27–31%) (Supplementary Information 1). Notably, NP-altered microbiomes exhibited greater representation of functions related to *microbial metabolism in diverse environments* and *biosynthesis of secondary metabolites*.

## Discussion

### NP-altered microbiomes enhance parasite proliferation in *D. magna*

Recipients of NP-altered microbiomes showed significantly increased *A. monospora* spore production, confirming that elevated parasite burdens previously observed in NP-exposed *Daphnia* [9, 27] are at least partially mediated by microbiome alteration.

Although our experimental design employed relatively high NP concentrations and parasite doses to ensure consistent microbiome alteration and infection, natural systems likely experience lower and more variable exposures [53, 54]. However, acute and chronic exposure to NPs and other types of nanoparticles at lower concentrations (e.g. NPs at 1 mg l^−1^ [55], silver nanoparticles at 0.4–0.7 mg l^−1^ [56]) produced mixed effects on the *Daphnia* microbiome, with some studies reporting significant alterations and others reporting nonsignificant changes. Nonetheless, the principle demonstrated here—that pollutant-induced microbiome shifts can amplify parasite proliferation—remains ecologically relevant, particularly in pollution hotspots.

The microbiota is widely recognized as a protective barrier that modulates host immunity and suppress pathogen colonization [57]. Stable microbial communities can enhance host defence [58], while dysbiosis—whether induced by pollutants, antibiotics, or immune disruption–often correlates with increased parasitism in humans, fish, and bivalves [59–61]. Similar patterns occur in Pacific oyster mortality syndrome [62] and chytrid infections in amphibians [63], underscoring microbiome stability as a key component of host resistance. Our results extend these findings by showing that anthropogenic nanoparticles alone can induce alternative microbiome states that are more permissive to parasite proliferation.

Mechanistically, several NP-induced taxonomic shifts are consistent with reduced protective capacity. Flavobacteriales and Enterobacterales, both depleted in recipients of NP-altered microbiomes, are integral to *Daphnia* health. Flavobacteriales often dominate *Daphnia* microbiomes [64] and contribute to antimicrobial defence [65], microbial community support [66], degradation of complex biomolecules [67], and parasite resistance [68]. *Flavobacterium psychrophilum* can modulate immune gene expression in fish, enhancing phagocytosis and antigen presentation [69]. Consistent with earlier work showing NP-induced reductions in Flavobacteriales [8], our study now demonstrates a causal connection between this NP-induced decline and downstream microbiome and host responses. Enterobacterales likewise support *Daphnia* growth and reproduction [70], nutrient provisioning [71], and pathogen defence in aquatic organisms [72]. In *Caenorhabditis elegans*, commensal *Pantoea* strains improve host fitness and protects against *Pseudomonas aeruginosa* colonization [73]. Thus, the depletion of these beneficial groups in the present study likely reduces protective functions within the microbiome.

In contrast, NP-altered microbiomes were enriched in Caulobacterales and Chitinophagales. Certain *Caulobacter* species are associated with infections in humans [74] and insects [75], suggesting that their enrichment (e.g. *Caulobacter*, *Brevidomonas*, and *Stagnimonas*) may reflect increased prevalence of opportunistic bacteria. Chitinophagales, known polysaccharide degraders [76] with potential antifungal activity [77], and Hyphomicrobiales, known nitrogen fixers [78] and metal oxidizers [79], may thrive in disturbed communities but appear less involved in host protection. Collectively, these compositional shifts may create a microbial environment that facilitates parasite establishment and proliferation.

Altered microbial communities can predispose hosts to infection [80] by modifying conditions that favour parasite colonization, replication, or virulence. Parasites and microbiota often engage in bidirectional interactions [81]. For example, increasing the abundance of a core honeybee gut microbe enhances susceptibility to the protozoan parasite *Lotmaria passim* [82], while viruses exploit gut bacteria to enhance replication and transmission in mice [83], and several microbial taxa can directly induce egg hatching of the parasitic nematode *Trichuris muris* [84]. Thus, NP-induced restructuring may not only weaken host resistance but also create microbial conditions that directly or indirectly enhance parasite growth.

Microbiomes also influence host digestion and metabolism [85]. By enhancing nutrient extraction, they may boost resistance to infection or provide parasites with additional resources to support their growth [86]. In *Daphnia*, the microbiome contributes to nutrient acquisition [31], suggesting that NP-induced shifts may modify nutrient availability in ways that favour parasite reproduction. While many studies focus on parasite-driven dysbiosis [64, 87], our findings demonstrate that pollutant-driven dysbiosis alone can facilitate heightened infection.

### Microbiome shifts compromise host reproduction but not survival

Recipients of NP-altered microbiomes exhibited significantly reduced fecundity, despite no detectable differences in survival. This aligns with life-history trade-offs in which reproduction is reduced under stress while survival is maintained [88]. Managing both dysbiosis and infection likely imposes energetic costs, reducing investment in offspring production. Similar reductions in reproductive output following microbiome disruption have been observed in *Aurelia aurita* under temperature and salinity stress [88] and in *Daphnia* exposed to toxigenic cyanobacteria [89]. Our findings support broader evidence that dysbiosis can impair growth and fecundity [90], highlighting the ecological relevance of microbiome-mediated shifts in keystone species such as *D. magna*.

### NP-altered microbiomes remained persistently distinct

NP-induced microbiome shifts persisted throughout host lifespan, even in the absence of direct NP exposure. This demonstrates that NP exposure can induce stable microbial community states rather than transient perturbations. While previous work showed that continuous NP exposure alters *Daphnia* microbiomes [8], our transplantation experiment demonstrates that these altered states remain distinct even after being transplanted into NP-free hosts.

Community divergence was associated with parasite spore output and host lifespan, and constrained ordination analyses confirmed that the treatment was a major driver of compositional shifts. Several mechanisms likely contribute to the stability of NP-altered microbiomes. First, loss of sensitive core taxa may disrupt foundational community structure [91], reducing resilience and limiting the ability to reassembly towards the original state. Second, enrichment of opportunistic or NP-resistant taxa may create novel microbial interactions [91] that reinforce the altered configuration. Third, NP-induced physiological changes in donors [92] may have shaped microbiome assembly trajectories that persist following transplantation even in the absence of pollutant exposure. Together, these effects may result in feedback loops reinforcing dysbiosis and impairing resilience to subsequent environmental or biotic stressors.

### Functional restructuring accompanies taxonomic shifts

Taxonomic differences were mirrored by broad changes in predicted functional potential. NP-altered microbiomes showed enrichment of pathways related to microbial metabolism in diverse environments and biosynthesis of secondary metabolites, indicating a shift towards metabolically versatile and environmentally responsive traits.

Several indicator taxa enriched in NP-altered microbiomes are associated with nitrogen cycling [78, 93], oxidation processes [79], denitrification [94], and hydrocarbon degradation [95], suggesting a reorganization of nutrient transformation pathways within the host-associated microbiomes. These taxa include *Bosea beijingensis* (ASV 60), *Oligoflexus tunisiensis* (ASV 52), *Gemmobacter* spp. (ASV 89), and *Sphingorhabdus rigui* (ASV 105).

Conversely, the depletion of *Burkholderiales*—known mutualists of *Daphnia* [30]—may comprise host-associated nutrient cycling and symbiotic functions. Representative taxa include *Rhodoferax aquaticus* (ASV 46), *Hydrogenophaga aromaticivorans* (ASV 194), and *Aquariibacter albus* (ASV 202). Members of this group are implicated in nitrogen and sulphur cycling [96], as well as degradation of organic pollutants [97, 98]. Their loss may therefore reduce microbiome-mediated support of the host, while simultaneously opening ecological niches that can be occupied by opportunistic taxa, potentially increasing susceptibility to infection.

NP-driven taxonomic shifts were accompanied by changes in predicted metabolic potential, including enrichment of pathways related to microbial metabolism in diverse environments and secondary metabolite biosynthesis. These results are consistent with reports that NPs alter *Daphnia* metabolism [99] and restructure microbial communities and their functional profiles [100]. While functional predictions based on 16S rRNA require cautious interpretation, the concordance between taxonomic changes and predicted metabolic shifts supports the conclusion that NP exposure drives coordinated alterations in community composition and function. Future studies using direct functional assays will be essential to validate these inferred pathways.

## Conclusions

This study reveals that NP-induced microbiome alterations can independently modulate host and parasite fitness. By employing microbiome transplantation, we disentangled indirect microbiome-mediated effects from direct nanoplastic toxicity. NP-altered microbiomes, characterized by depletion of beneficial taxa and enrichment of stress-tolerant or opportunistic bacteria, promoted increased parasite reproduction and reduced host fecundity without affecting survival. These shifts persisted across the host lifespan and were accompanied by broad functional restructuring, suggesting that pollutant exposure can generate stable alternative microbiome states with ecological consequences. Given the keystone role of *Daphnia* in freshwater ecosystems, microbiome-mediated responses to nanoplastics may influence not only individual fitness but also parasite transmission dynamics and broader ecological processes. Our findings highlight that emerging pollutants exert ecological effects not only through direct toxicity but also via restructuring of host-associated microbial communities. Integrating microbiome-mediated pathways into ecological risk assessments will be essential for forecasting the long-term biological consequences of increasing plastic pollution in aquatic systems.

## Supplementary Material

Supplementary_Material_ycag109

## Data Availability

The processed 16S sequencing data is available in the National Centre for Biotechnology Information—Sequence Read Archive (NCBI-SRA) under accession numbers SAMN48680537 through SAMN48680628 (BioProject accession number PRJNA1266608). Dataset and R scripts are available in Zenodo repository (10.5281/zenodo.18745189).
