# Supplementary material for: Nanoplastic-induced microbiome shifts reduce Daphnia fitness and increase parasite reproduction
Source: ISME Commun. 2026 Apr 20;6(1):ycag109. doi: 10.1093/ismeco/ycag109 (PMC13196593; doi:10.1093/ismeco/ycag109)
Supplement: Supplementary_Material_ycag109 [file supplementary_material_ycag109.zip › Supplementary Figure Labels.docx]

**Figure S1.** Assessment of bacterial contamination in media and egg sources used to generate axenic *Daphnia*. (**A**) Bacterial growth curves over four weeks, based on optical density (OD_600_). Treatment groups included axenic eggs, sterile SSS medium, non-axenic eggs, and non-sterile SSS medium inoculated in LB medium. The x-axis represents time (weeks), and the y-axis shows OD_600_ values. Each data point represents an individual OD_600_ measurement (6 replicates, 3 technical measurements per replicate). Bold larger icons indicate mean OD_600_ values ± standard error. (**B**) Bacterial growth after four weeks, showing visible turbidity differences across inoculation sources. Sterile LB medium served as a reference.

**Figure S2.** Relative abundance of bacterial orders in negative controls (DNA extraction and PCR blanks). Orders representing <1% of total reads and not present across all samples are grouped as ‘Other’, consistent with the classification applied to experimental samples (Fig. 5). Negative controls are dominated by rare taxa (Other), Burkholderiales, and Pseudomonadales. The absence of other dominant bacterial orders confirms that biological sample microbiomes are distinct from contamination sources.

**Figure S3.** Alpha and beta diversity of microbiomes from NP-exposed and control donors and recipients. (**A**) ASV richness and (**B**) Inverse Simpson Index represent alpha diversity, reflecting the number of ASVs and community evenness, respectively. Green box plots indicate control microbiomes, red box plots NP-altered microbiomes, with donors bordered by dashed line and recipients by solid line. (**C**) Jaccard dissimilarity and (**D**) Bray-Curtis dissimilarity depicts beta diversity, illustrating differences in community composition. Light colours represent donors, dark colours recipients. NP-altered donors and recipients cluster separately from controls, indicating that NP exposure significantly alters the *Daphnia* microbiome, and these changes are maintained after transplantation into axenic hosts (after 48 hours).

**Figure S4**. Alpha diversity of recipient microbiomes over host lifespan post-infection. ASV richness (left) and Inverse Simpson Index (right) are plotted for *Daphnia* transplanted with control or NP-altered microbiomes. Each point represents an individual, with linear trends and 95% confidence intervals shown (green – control; red – NP-altered microbiome). In the NP-altered microbiomes, both richness and evenness displayed a stronger positive relationship with host lifespan, suggesting increased taxonomic accumulation and a shift toward more even community structure over time.

**Figure S5.** Heatmap of log-transformed counts of indicator ASVs differentiating control and NP-altered microbiomes in recipients. Rows represent ASVs with their corresponding bacterial orders, and columns represent individual host samples grouped by treatment groups. **Yellow** shading indicates ASVs uniquely present, and **gray** shading indicates ASVs absent in NP-altered microbiomes compared to controls. Identities and putative ecological roles of highlighted indicator ASVs are presented in Table S5.

**Figure S6.** **Nanoplastic exposure alters predicted microbiome functional profiles.** (**A**) Volcano plot of predicted KEGG Ortholog (KO) abundances comparing NP-altered and control microbiomes. (**B**) Volcano plot of predicted Enzyme Commission (EC) abundances. Points represent individual features; red and green indicate enrichment in NP-altered and control microbiomes, respectively, and grey indicates non-significant features (FDR-adjusted p < 0.05; dashed line). (**C**) Principal component analysis (PCA) of log2-transformed KO abundances. (**D**) PCA of variance-stabilized EC abundances. Points represent individual microbiome samples; colours denote treatment group. Percent variance explained by each principal component is shown on the axes.
