# Supplementary material for: Nanoplastic-induced microbiome shifts reduce Daphnia fitness and increase parasite reproduction
Source: ISME Commun. 2026 Apr 20;6(1):ycag109. doi: 10.1093/ismeco/ycag109 (PMC13196593; doi:10.1093/ismeco/ycag109)
Supplement: Supplementary_Material_ycag109 [file supplementary_material_ycag109.zip › Supplementary Tables.docx]

**Table S1.** Type II ANOVA results for alpha diversity metrics in microbiomes of *Daphnia* donors and recipients of control and NP-altered microbiomes: (**A**) ASV richness and (**B**) Inverse Simpson Index. Pairwise comparisons were performed using estimated marginal means (emmeans), with p-values corrected for multiple testing using the false discovery rate (FDR) method. Significant differences [ Pr(>Chisq) or p < 0.05] are shown in bold.

|  |  |  |  |  |  |
| --- | --- | --- | --- | --- | --- |
| **A** ASV Richness | | | | | |
| Analysis of deviance (Type II test): Chisq = 10.384, df = 3 , p-value [Pr(>Chisq)] = **0.016** | | | | | |
| Pairwise comparisons | | | | | |
| Contrast | Estimate | SE | df | z ratio | p-value |
| **control donor vs. NP-altered donor** | -0.377 | 0.131 | inf | -2.886 | **0.023** |
| control donor vs. control recipient | -0.027 | 0.152 | inf | -0.176 | 0.860 |
| control donor vs. NP-altered recipient | -0.103 | 0.150 | inf | -0.685 | 0.740 |
| NP-altered donor vs. control recipient | 0.350 | 0.147 | inf | 2.387 | 0.051 |
| NP-altered donor vs. NP-altered recipient | 0.274 | 0.145 | inf | 1.892 | 0.117 |
| control recipient vs. NP-altered recipient | -0.076 | 0.164 | inf | -0.463 | 0.772 |
|  |  |  |  |  |  |
| **B** Inverse Simpson Index | | | | | |
| Analysis of deviance (Type II test): Chisq = 3.070, df = 3 , p-value (Pr(>Chisq) = 0.381 | | | | | |
| Pairwise comparisons | | | | | |
| Contrast | Estimate | SE | df | z ratio | p-value |
| control donor vs. NP-altered donor | -0.124 | 0.193 | 16 | -0.639 | 0.638 |
| control donor vs. control recipient | -0.280 | 0.216 | 16 | -1.296 | 0.638 |
| control donor vs. NP-altered recipient | -0.337 | 0.216 | 16 | -1.557 | 0.638 |
| NP-altered donor vs. control recipient | -0.157 | 0.216 | 16 | 0.724 | 0.638 |
| NP-altered donor vs. NP-altered recipient | -0.213 | 0.216 | 16 | -0.985 | 0.638 |
| control recipient vs. NP-altered recipient | -0.056 | 0.237 | 16 | -0.238 | 0.815 |

**Table S2.** PERMANOVAs and pairwise comparison results for beta diversity metrics in microbiomes of *Daphnia* donors and recipients of control and NP-altered microbiomes: (**A**) Jaccard Dissimilarity and (**B**) Bray-Curtis Dissimilarity. Analyses were performed using 9999 permutations, and p-values for multiple pairwise comparisons were FDR-corrected. Significant differences (p: Pr(>F) < 0.05) are shown in bold.

|  | | | | | | |
| --- | --- | --- | --- | --- | --- | --- |
| **A** Jaccard Dissimilarity | | | | | | |
| Permutation test | | | | | | |
|  | | df | SumOfSqs | R^2^ | F | p-value Pr(>F) |
| **Model** | | 3 | 1.940 | 0.303 | 2.321 | **1e-04** |
| Residual | | 16 | 4.457 | 0.697 |  |  |
| Total | | 19 | 6.397 | 1.000 |  |  |
|  | | | | | | |
| Pairwise comparisons | | | | | | |
|  |  | df | SumOfSqs | R^2^ | F | p-value adj Pr(>F) |
| **control donor vs.**  **NP-altered donor** | Model | 1 | 0.739 | 0.207 | 2.606 | **0.014** |
|  | Residual | 10 | 2.834 | 0.793 |  |  |
|  | Total | 11 | 3.573 | 1.000 |  |  |
|  |  |  |  |  |  |  |
| **control donor vs.**  **control recipient** | Model | 1 | 0.448 | 0.180 | 1.701 | **0.014** |
|  | Residual | 8 | 2.037 | 0.820 |  |  |
|  | Total | 9 | 2.485 | 1.00 |  |  |
|  |  |  |  |  |  |  |
| **control donor vs.**  **NP-altered recipient** | Model | 1 | 0.806 | 0.274 | 3.020 | **0.014** |
|  | Residual | 8 | 2.136 | 0.726 |  |  |
|  | Total | 9 | 2.942 | 1.000 |  |  |
|  |  |  |  |  |  |  |
| **NP-altered donor vs.**  **control recipient** | Model | 1 | 0.631 | 0.214 | 2.174 | **0.014** |
|  | Residual | 8 | 2.321 | 0.786 |  |  |
|  | Total | 9 | 2.952 | 1.000 |  |  |
|  |  |  |  |  |  |  |
| **NP-altered donor vs.**  **NP-altered recipient** | Model | 1 | 0.589 | 0.196 | 1.947 | **0.018** |
|  | Residual | 8 | 2.420 | 0.804 |  |  |
|  | Total | 9 | 3.009 | 1.000 |  |  |
|  |  |  |  |  |  |  |
| **control recipient vs.**  **NP-altered recipient** | Model | 1 | 0.649 | 0.286 | 2.340 | **0.030** |
|  | Residual | 6 | 1.623 | 0.714 |  |  |
|  | Total | 7 | 2.272 | 1.000 |  |  |
|  | | | | | | |
| **B** Bray-Curtis Dissimilarity | | | | | | |
| Permutation test | | | | | | |
|  | | df | SumOfSqs | R^2^ | F | p-value Pr(>F) |
| **Model** | | 3 | 1.390 | 0.443 | 4.248 | **1e-04** |
| Residual | | 16 | 1.745 | 0.557 |  |  |
| Total | | 19 | 3.135 | 1.000 |  |  |
|  | |  |  |  |  |  |
| Pairwise comparisons | | | | | | |
|  |  | df | SumOfSqs | R^2^ | F | p-value adj Pr(>F) |
| **control donor vs.**  **NP-altered donor** | Model | 1 | 0.557 | 0.319 | 4.680 | **0.011** |
|  | Residual | 10 | 1.191 | 0.681 |  |  |
|  | Total | 11 | 1.748 | 1.000 |  |  |
|  |  |  |  |  |  |  |
| **control donor vs.**  **control recipient** | Model | 1 | 0.276 | 0.258 | 2.779 | **0.011** |
|  | Residual | 8 | 0.796 | 0.742 |  |  |
|  | Total | 9 | 1.072 | 1.000 |  |  |
|  |  |  |  |  |  |  |
| **control donor vs.**  **NP-altered recipient** | Model | 1 | 0.606 | 0.409 | 5.540 | **0.013** |
|  | Residual | 8 | 0.874 | 0.591 |  |  |
|  | Total | 9 | 1.480 | 1.000 |  |  |
|  |  |  |  |  |  |  |
| **NP-altered donor vs.**  **control recipient** | Model | 1 | 0.433 | 0.333 | 3.986 | **0.011** |
|  | Residual | 8 | 0.870 | 0.667 |  |  |
|  | Total | 9 | 1.303 | 1.000 |  |  |
|  |  |  |  |  |  |  |
| **NP-altered donor vs.**  **NP-altered recipient** | Model | 1 | 0.423 | 0.309 | 3.570 | **0.013** |
|  | Residual | 8 | 0.949 | 0.691 |  |  |
|  | Total | 9 | 1.372 | 1.000 |  |  |
|  |  |  |  |  |  |  |
| **control recipient vs.**  **NP-altered recipient** | Model | 1 | 0.465 | 0.456 | 5.036 | **0.027** |
|  | Residual | 6 | 0.554 | 0.544 |  |  |
|  | Total | 7 | 1.019 | 1.000 |  |  |

**Table S3.** Generalized Linear Models (GLM) results for alpha diversity metrics in *Daphnia* microbiomes between recipient treatment groups (control vs. NP-altered): (**A**) ASV richness and (**B**) Inverse Simpson Index. Significant effects (p: Pr(>|z|) or Pr(>|t|) < 0.05) are shown in bold.

|  | | | | |
| --- | --- | --- | --- | --- |
| **A** ASV Richness | | | | |
|  | Estimate | Std. error | z value  (Poisson model) | p-value Pr(>\|z\|) |
| **(intercept)** | 3.907 | 0.195 | 20.024 | **<2e-16** |
| **Treatment** | -0.537 | 0.221 | -2.424 | **0.015** |
| Days lived post infection | 0.009 | 0.012 | 0.769 | 0.442 |
| **Treatment x Days lived post infection** | 0.037 | 0.013 | 2.791 | **0.005** |
| Null deviance: 138.908 on df = 66, Residual deviance: 63.982 on df = 63, AIC = 471.26 | | | | |
|  | | | | |
| **B** Inverse Simpson Index | | | | |
|  | Estimate | Std. error | z value  (Gamma model) | p-value Pr(>\|t\|) |
| **(intercept)** | 2.130 | 0.685 | 3.109 | **0.003** |
| Treatment | -1.481 | 0.775 | -1.911 | 0.061 |
| Days lived post infection | -0.031 | 0.042 | -0.737 | 0.464 |
| **Treatment x Days lived post infection** | 0.114 | 0.047 | 2.407 | **0.019** |
| Null deviance: 21.028 on df = 66, Residual deviance: 14.762 on df = 63, AIC = 343.62 | | | | |

**Table S4.** Taxonomic classification of ASVs identified as Indicator ASVs by treatment. ASVs were classified up to the genus level when possible to identify taxa associated with treatment effects and the interactions with host survival duration post-infection.

| **ASV** | **Kingdom** | **Phylum** | **Class** | **Order** | **Family** | **Genus** |
| --- | --- | --- | --- | --- | --- | --- |
| ASV_1 | Bacteria | Pseudomonadota | Gammaproteobacteria | Pseudomonadales | Pseudomonadaceae | *Pseudomonas* |
| ASV_3 | Bacteria | Pseudomonadota | Gammaproteobacteria | Pseudomonadales | Pseudomonadaceae | *Pseudomonas* |
| ASV_4 | Bacteria | Pseudomonadota | Gammaproteobacteria | Burkholderiales | Comamonadaceae | *Inhella* |
| ASV_7 | Bacteria | Bacteroidota | Bacteroidia | Cytophagales | Spirosomataceae | *Emticicia* |
| ASV_9 | Bacteria | Pseudomonadota | Gammaproteobacteria | Burkholderiales | Comamonadaceae | *Hydrogenophaga* |
| ASV_10 | Bacteria | Pseudomonadota | Gammaproteobacteria | Burkholderiales | Comamonadaceae | Unknown |
| ASV_11 | Bacteria | Pseudomonadota | Gammaproteobacteria | Burkholderiales | Rhodocyclaceae | *Methyloversatilis* |
| ASV_12 | Bacteria | Bacteroidota | Bacteroidia | Flavobacteriales | Flavobacteriaceae | *Flavobacterium* |
| ASV_13 | Bacteria | Bacteroidota | Bacteroidia | Chitinophagales | Chitinophagaceae | Unknown |
| ASV_15 | Bacteria | Pseudomonadota | Gammaproteobacteria | Enterobacterales | Alteromonadaceae | *Rheinheimera* |
| ASV_16 | Bacteria | Bacteroidota | Bacteroidia | Cytophagales | Spirosomataceae | *Runella* |
| ASV_17 | Bacteria | Pseudomonadota | Gammaproteobacteria | Burkholderiales | Oxalobacteraceae | *Undibacterium* |
| ASV_20 | Bacteria | Pseudomonadota | Gammaproteobacteria | Enterobacterales | Alteromonadaceae | *Rheinheimera* |
| ASV_22 | Bacteria | Pseudomonadota | Alphaproteobacteria | Hyphomicrobiales | Devosiaceae | *Devosia* |
| ASV_23 | Bacteria | Myxococcota | Polyangiia | Polyangiales | Polyangiaceae | *Pajaroellobacter* |
| ASV_25 | Bacteria | Pseudomonadota | Alphaproteobacteria | Reyranellales | Reyranellaceae | *Reyranella* |
| ASV_26 | Bacteria | Bacteroidota | Bacteroidia | Cytophagales | Spirosomataceae | *Lacihabitans* |
| ASV_29 | Bacteria | Pseudomonadota | Alphaproteobacteria | Unknown | Unknown | Unknown |
| ASV_30 | Bacteria | Bacteroidota | Bacteroidia | Chitinophagales | Saprospiraceae | Unknown |
| ASV_34 | Bacteria | Pseudomonadota | Gammaproteobacteria | Burkholderiales | Comamonadaceae | *Ideonella* |
| ASV_35 | Bacteria | Pseudomonadota | Alphaproteobacteria | Hyphomicrobiales | Rhizobiaceae | *Shinella* |
| ASV_36 | Bacteria | Bacteroidota | Bacteroidia | Chitinophagales | Unknown | Unknown |
| ASV_37 | Bacteria | Bacteroidota | Bacteroidia | Sphingobacteriales | Sphingobacteriaceae | *Solitalea* |
| ASV_39 | Bacteria | Bacteroidota | Bacteroidia | Flavobacteriales | Flavobacteriaceae | *Flavobacterium* |
| ASV_44 | Bacteria | Pseudomonadota | Gammaproteobacteria | Burkholderiales | Burkholderiaceae | *Limnobacter* |
| ASV_45 | Bacteria | Bacteroidota | Bacteroidia | Flavobacteriales | Crocinitomicaceae | *Fluviicola* |
| ASV_46 | Bacteria | Pseudomonadota | Gammaproteobacteria | Burkholderiales | Comamonadaceae | *Rhodoferax* |
| ASV_48 | Bacteria | Pseudomonadota | Alphaproteobacteria | Caulobacterales | Caulobacteraceae | *Caulobacter* |
| ASV_49 | Bacteria | Bacteroidota | Bacteroidia | Sphingobacteriales | NS11-12 marine group | Unknown |
| ASV_51 | Bacteria | Bacteroidota | Bacteroidia | Flavobacteriales | Unknown | Unknown |
| ASV_52 | Bacteria | Bdellovibrionota | Oligoflexia | Oligoflexales | Oligoflexaceae | *Oligoflexus* |
| ASV_53 | Bacteria | Bacteroidota | Bacteroidia | Sphingobacteriales | NS11-12 marine group | Unknown |
| ASV_56 | Bacteria | Bacteroidota | Bacteroidia | Chitinophagales | Saprospiraceae | Unknown |
| ASV_58 | Bacteria | Armatimonadota | Armatimonadia | Armatimonadales | Armatimonadaceae | *Armatimonas* |
| ASV_60 | Bacteria | Pseudomonadota | Alphaproteobacteria | Hyphomicrobiales | Beijerinckiaceae | *Bosea* |
| ASV_62 | Bacteria | Bdellovibrionota | Bdellovibrionia | Bdellovibrionales | Pseudobdellovibrionaceae | *Bdellovibrio* |
| ASV_65 | Bacteria | Bacteroidota | Bacteroidia | Sphingobacteriales | Sphingobacteriaceae | *Arcticibacter* |
| ASV_68 | Bacteria | Pseudomonadota | Alphaproteobacteria | Sphingomonadales | Sphingomonadaceae | *Sphingorhabdus* |
| ASV_69 | Bacteria | Actinomycetota | Actinobacteria | Micrococcales | Microbacteriaceae | *Candidatus Aquiluna* |
| ASV_72 | Bacteria | Pseudomonadota | Alphaproteobacteria | Caulobacterales | Caulobacteraceae | *Caulobacter* |
| ASV_76 | Bacteria | Actinomycetota | Actinobacteria | Micrococcales | Microbacteriaceae | *Galbitalea* |
| ASV_78 | Bacteria | Pseudomonadota | Alphaproteobacteria | Caulobacterales | Caulobacteraceae | *Brevundimonas* |
| ASV_81 | Bacteria | Pseudomonadota | Alphaproteobacteria | Caulobacterales | Caulobacteraceae | *Caulobacter* |
| ASV_83 | Bacteria | Pseudomonadota | Alphaproteobacteria | Rhodobacterales | Paracoccaceae | Unknown |
| ASV_84 | Bacteria | Bacteroidota | Bacteroidia | Chitinophagales | Chitinophagaceae | *Sediminibacterium* |
| ASV_87 | Bacteria | Pseudomonadota | Alphaproteobacteria | Rhodobacterales | Paracoccaceae | *Fuscovulum* |
| ASV_89 | Bacteria | Pseudomonadota | Alphaproteobacteria | Rhodobacterales | Paracoccaceae | *Gemmobacter* |
| ASV_90 | Bacteria | Pseudomonadota | Gammaproteobacteria | Burkholderiales | Comamonadaceae | Unknown |
| ASV_96 | Bacteria | Pseudomonadota | Gammaproteobacteria | Pseudomonadales | Moraxellaceae | *Perlucidibaca* |
| ASV_105 | Bacteria | Pseudomonadota | Alphaproteobacteria | Sphingomonadales | Sphingomonadaceae | *Sphingorhabdus* |
| ASV_107 | Bacteria | Pseudomonadota | Gammaproteobacteria | Burkholderiales | Comamonadaceae | Unknown |
| ASV_110 | Bacteria | Candidatus Kapabacteria | Kapabacteria | Kapabacteriales | Unknown | Unknown |
| ASV_114 | Bacteria | Pseudomonadota | Gammaproteobacteria | Burkholderiales | Comamonadaceae | *Hydrogenophaga* |
| ASV_115 | Bacteria | Pseudomonadota | Gammaproteobacteria | Salinisphaerales | Nevskiaceae | *Stagnimonas* |
| ASV_117 | Bacteria | Bacteroidota | Bacteroidia | Chitinophagales | Saprospiraceae | Unknown |
| ASV_118 | Bacteria | Pseudomonadota | Alphaproteobacteria | Paracaedibacterales | Paracaedibacteraceae | Unknown |
| ASV_121 | Bacteria | Pseudomonadota | Alphaproteobacteria | Caulobacterales | Caulobacteraceae | *Caulobacter* |
| ASV_129 | Bacteria | Pseudomonadota | Gammaproteobacteria | Burkholderiales | Rhodocyclaceae | *Dechloromonas* |
| ASV_132 | Bacteria | Pseudomonadota | Alphaproteobacteria | Caulobacterales | Caulobacteraceae | *Brevundimonas* |
| ASV_139 | Bacteria | Pseudomonadota | Gammaproteobacteria | Salinisphaerales | Nevskiaceae | *Nevskia* |
| ASV_149 | Bacteria | Pseudomonadota | Gammaproteobacteria | Salinisphaerales | Nevskiaceae | *Stagnimonas* |
| ASV_155 | Bacteria | Pseudomonadota | Alphaproteobacteria | Sphingomonadales | Sphingomonadaceae | *Sphingobium* |
| ASV_162 | Bacteria | Pseudomonadota | Alphaproteobacteria | Caulobacterales | Caulobacteraceae | *Caulobacter* |
| ASV_183 | Bacteria | Pseudomonadota | Alphaproteobacteria | Hyphomicrobiales | Hyphomicrobiaceae | *Hyphomicrobium* |
| ASV_189 | Bacteria | Pseudomonadota | Gammaproteobacteria | Salinisphaerales | Nevskiaceae | *Hydrocarboniphaga* |
| ASV_193 | Bacteria | Pseudomonadota | Alphaproteobacteria | Paracaedibacterales | Paracaedibacteraceae | Unknown |
| ASV_194 | Bacteria | Pseudomonadota | Gammaproteobacteria | Burkholderiales | Comamonadaceae | *Hydrogenophaga* |
| ASV_195 | Bacteria | Pseudomonadota | Alphaproteobacteria | Sphingomonadales | Sphingomonadaceae | *Parablastomonas* |
| ASV_202 | Bacteria | Pseudomonadota | Gammaproteobacteria | Burkholderiales | Comamonadaceae | Unknown |
| ASV_251 | Bacteria | Pseudomonadota | Alphaproteobacteria | Hyphomicrobiales | Rhizobiaceae | *Mesorhizobium* |
| ASV_376 | Bacteria | Actinomycetota | Actinobacteria | Frankiales | Sporichthyaceae | *hgcI clade* |

**Table S5.** Identities and putative ecological roles of indicator ASVs either present (yellow highlight) or absent (gray highlight) in the NP-altered microbiome recipients compared to the control microbiome recipients (Figure S5). Taxonomic classifications are based on SILVA assignments, and closest matches were identified using 100% NCBI BLAST results. A ≥99.5% identity threshold was required for species-level assignment; sequences below this threshold was conservatively assigned at the genus level (e.g., *Genus* spp.). Taxa with limited or unresolved classification were labelled as “uncultured”. Ecological roles were inferred from literature associated with the closest matched strains.

| **ASV** | **Taxonomic Classification** | **Closest known BLAST hit**  (100% identity; cut-off 99.5%) | **Putative functional ecology** |
| --- | --- | --- | --- |
|  |  |  |  |
| **ASV_20** | Order: Enterobacterales  Family: Alteromonadaceae | *Rheinheimera riviphila* [1] | *Rheinheimera* spp. are known to produce compounds against ciliates and damage their symbiotic bacteria, suggesting strong antimicrobial activity [2], and are able to carry mobile genetic element that gives resistance to antibiotics and arsenic [3]. |
|  |  |  |  |
| **ASV_46** | Order: Burkholderiales  Family: Comamonadaceae | *Rhodoferax aquaticus* [4] | *R. aquaticus* contain GS/GOGOAT pathway-related genes associated with nitrate ammonification [4]. Other *Rhodoferax* species were reported to have a strong capacity for nitrogen fixation, sulfur oxidation, and multidrug efflux pump mechanisms [5, 6]. |
|  |  |  |  |
| **ASV_52** | Order: Oligoflexales  Family: Oligoflexaceae | *Oligoflexus tunisiensis* [7] | *O. tunisiensis* contains gene related to nitrous oxide (NO2) producing activity [7]. |
|  |  |  |  |
| **ASV_60** | Order: Hyphomicrobiales Family: Beijerinckiaceae | *Bosea beijingensis* [8] | *Bosea* spp. contain genes related to nitrogen fixation and oxidation of arsenic, sulfur, and antimony [9–11]. |
|  |  |  |  |
| **ASV_72** | Order: Caulobacterales  Family: Caulobacteraceae | *Caulobacter rhizosphaerae* [12]  *Caulobacter soli* [13]  *Caulobacter henricii* [14] | *Caulobacter* sp. are common in aquatic environment and some species along with other members of Alphaproteobacteria thrive at the water and air interfaces via creation of surface structures which may function to facilitate colonization in the air-liquid boundaries [15]. |
| **ASV_81** | Order: Caulobacterales Family: Caulobacteraceae | *Caulobacter* spp. |  |
|  |  |  |  |
| **ASV_89** | Order: Rhodobacterales Family: Paracoccaceae | *Gemmobacter denitrificans* [16]  *Gemmobacter lanyuensis* [17]  *Gemmobacter caeruleus* [18]  *Gemmobacter aquatilis* [19, 20] | *Gemmobacter* strains were found to contain genes for the oxidation of alkane to carbon dioxide and nitrate reduction [21]. *G. denitrificans* isolated from pond water is a denitrifying bacterium removing nitrites and nitrates [16].  *G. lanyuensis* and *G. caeruleus* were isolated from a freshwater spring [17] and lake interfacial sediment [18], respectively. Currently, no reports regarding the putative functional role for these strains.  *G. aquatilis* isolated from a pond were determined to have functional genes for methylamine utilization [22]. |
|  |  |  |  |
| **ASV_105** | Order: Sphingomonadales  Family: Sphingomonadaceae | *Sphingorhabdus rigui* [23] | The family Sphingomonadaceae includes bacterial strains used for bioremediation applications [24]. *Sphingorhabdus* strains were reported to have hydrocarbon-degrading capabilities [25] and one strain (*Sphingorhabdus* sp. YGSMI21) showed ability to optically decompose (R)-styrene oxide (SO) and serve as biocatalyst in the production of various (S)-CSO [26]. |
|  |  |  |  |
| **ASV_107** | Order: Burkholderiales Family: Comamonadaceae | *Aquabacterium pictum* [27] | *A. pictum* was isolated from a river biofilm [27]. No functional ecological roles or applications are currently reported. |
|  |  |  |  |
| **ASV_110** | Order: Kapabacteria Family: Kapabacteriales | uncultured Kapabacteria | Genus Candidatus *Kapabacteria* have the potential nitrate removal [28]. |
|  |  |  |  |
| **ASV_114** | Order: Burkholderiales Family: Comamonadaceae | *Hydrogenophaga bisanensis* [29] | *H. bisanensis* was isolated from textile dye works wastewater [29]. Some *Hydrogenophaga* spp. are able to oxidize acetate in cooperation with other bacteria [30], potential enzyme activity for toxic compounds [31], and contain genes associated with chemolithoautotrophy, including CO2 fixation, H2 oxidation, S-compound oxidation, and denitrification [32].The family Comamonadaceae belonging to the order Burkholderiales includes bacteria that are organotrophs, denitrifiers and Iron(III)-reducing bacteria and hydrogen oxidizers [33]. |
|  |  |  |  |
| **ASV_117** | Order: Chitinophagales Family: Saprospiraceae | *Phaeodactylibacter* spp. | Members of the Saprospiraceae family are commonly found in aquatic environment and activated sludge for wastewater treatment and are known to hydrolyze and utilize complex carbon sources and proteins with some strains associated with predation of other bacteria and algae [34, 35]. |
| **ASV_118** | Order: Paracaedibacterales Family: Paracaedibacteraceae | Candidatus *Bodocaedibacter vickermanii*  [36] | Paracaedibacteraceae family includes strains known as endosymbionts of freshwater protists [37]. |
|  |  |  |  |
| **ASV_121** | Order: Caulobacterales Family: Caulobacteraceae | *Phenylobacterium ferrooxidans*  [38] | *P. ferrooxidans* is metabolically versatile and able to perform Fe oxidation [38]. |
|  |  |  |  |
| **ASV_129** | Order: Burkholderiales Family: Rhodocyclaceae | *Dechloromonas hankyongensis* [39] | *D. hankyongensis* is first isolated from a wetland in Korea [39]. Strains belonging to the genus *Dechloromonas* are associated to degrade xenobiotics [40]. |
|  |  |  |  |
| **ASV_132** | Order: Caulobacterales Family: Caulobacteraceae | *Brevundimonas alba* [14, 41] | Strains from the *Brevundimonas* genus are known for their denitrification abilities and resistance to metalloid oxyanions [42, 43]. |
|  |  |  |  |
| **ASV_139** | Order: Salinisphaerales Family: Nevskiaceae | *Nevskia ramosa* DSM 11499 | *N. ramosa* is commonly found in biofilm communities forming hydrophobic rosettes which are effective at facilitating attachment to PCBs (polychlorinated biphenyls) oil droplets [44]. Some *Nevskia* strains are also able to assimilate alkanes and utilize organic substrates [45]. |
|  |  |  |  |
| **ASV_149** | Order: Salinisphaerales Family: Nevskiaceae | *Stagnimonas* spp. | Aside from the *Nevskia* strains, members of the Nevskiaceae family showed abilities to utilize sulphate for energy metabolism [46] and degrade steroidal hormones [47]. |
|  |  |  |  |
| **ASV_193** | Order: Paracaedibacterales Family: Paracaedibacteraceae | uncultured Paracaedibacterales | Paracaedibacteraceae family includes strains known as endosymbionts of freshwater protists [37]. |
| **ASV_194** | Order: Burkholderiales Family: Comamonadaceae | *Hydrogenophaga aromaticivorans*  [48] | *H. aromaticivorans* is a para- and meta-xylene-degrading bacteria [48]. Members of this genus shows potential to degrade benzene in petroleum hydrocarbon contaminated environment [49]. |
|  |  |  |  |
| **ASV_195** | Order: Sphingomonadales Family: Sphingomonadaceae | *Novosphingobium fuchskuhlense*  [50] | Strain from the *Novosphingobium* genus can degrade organophosphate pesticides [51], estradiol [52], and aromatic compounds including high molecular mass polycyclic aromatic compounds [53, 54]. |
|  |  |  |  |
| **ASV_202** | Order: Burkholderiales Family: Comamonadaceae | *Aquariibacter albus* [55] | *A. albus* was isolated from a freshwater aquarium [55]. Belonging to the Order Burkholderiales, genera from this order can survive and degrade methyl tert-butyl ether [56] and are known symbionts of *Daphnia* [57–59] |
|  |  |  |  |
| **ASV_251** | Order: Hyphomicobiales Family: Rhizobiaceae | *Mesorhizobium* spp. | *Mesorhizobium* strains are common legume symbionts known for their denitrification abilities [60] and involvement in the nodulation processes of plants [61]. |
|  |  |  |  |
| **ASV_376** | Order: Frankiales Family: Sporichthyaceae | hgcl clade / uncultured *Longivirga* spp. | Actinobacteria are present and adaptable to most environment types and are known to play key environmental roles involved in decomposition of organic matter, bioremediation and bio weathering applications [62, 63]. |

**Table S6.** Compositional means (%) and standard deviations of dominant bacterial orders in *Daphnia* microbiomes across recipient treatment groups (control and NP-altered) for: (**A**) Overall differential abundances, (**B**) Differential abundances at 15 days post-infection, (**C**) at 17 days post-infection, (**D**) at 20 days post-infection. Significant differences in relative abundance were determined using Wald tests with FDR correction. Bacterial orders showing significant differences (padj < 0.05), are highlighted in bold.

|  | | | | | |
| --- | --- | --- | --- | --- | --- |
| **A** Overall total differential abundances between treatment group | | | | | |
| Order | %compositional mean ± standard deviation | | log2FoldChange | Wald Stat | padj |
|  | Control | NP-altered |  |  |  |
| Burkholderiales | 22.630 ± 12.313 | 33.968 ± 14.420 | 0.103 | 0.640 | 0.522 |
| **Caulobacterales** | **0.131 ± 0.126** | **0.979 ± 0.878** | 2.504 | 9.849 | **3.81e-22** |
| **Chitinophagales** | **0.288 ± 0.631** | **4.319 ± 3.102** | 3.582 | 10.945 | **7.69e-27** |
| Cytophagales | 7.202 ± 4.231 | 12.551 ± 7.100 | 0.224 | 1.114 | 0.292 |
| **Enterobacterales** | **3.206 ± 5.175** | **2.304 ± 7.190** | -2.175 | -4.903 | **2.08e-06** |
| **Flavobacteriales** | **16.541 ± 9.880** | **7.365 ± 8.036** | -1.830 | -4.977 | **1.79e-06** |
| **Hyphomicrobiales** | **1.043 ± 0.596** | **2.911 ± 2.073** | 0.877 | 4.976 | **1.79e-06** |
| **Paracaedibacterales** | **0.535 ± 0.476** | **0.403 ± 0.471** | -1.054 | -3.992 | **1.03e-04** |
| Pseudomonadales | 38.133 ± 14.589 | 26.405 ± 21.833 | -0.653 | -1.578 | 0.140 |
| **Reyranellales** | **0.455 ± 0.501** | **1.171 ± 1.289** | 0.749 | 2.253 | **0.033** |
| **Other** | **9.837 ± 3.797** | **7.624 ± 5.924** | -1.003 | -4.115 | **7.11e-05** |
|  | | | | | |
| **B** Differential abundances at 15 days post-infection | | | | | |
| Order | %compositional mean ± standard deviation | | log2FoldChange | Wald Stat | padj |
|  | Control | NP-altered |  |  |  |
| Burkholderiales | 17.817 ± 9.572 | 38.100 ± 14.164 | 0.574 | 1.961 | 0.078 |
| **Caulobacterales** | **0.095 ± 0.073** | **0.721 ± 0.345** | 2.372 | 4.686 | **1.5e-05** |
| **Chitinophagales** | **0.198 ± 0.279** | **4.697 ± 4.038** | 4.037 | 5.576 | **2.7e-07** |
| Cytophagales | 6.939 ± 3.591 | 9.423 ± 3.688 | -0.102 | -0.298 | 0.786 |
| **Enterobacterales** | **2.809 ± 2.157** | **0.513 ± 0.350** | -3.299 | -3.753 | **6.41e-04** |
| **Flavobacteriales** | **23.664 ± 10.892** | **8.611 ± 10.865** | -1.938 | -3.058 | **0.006** |
| Hyphomicrobiales | 0.845 ± 0.426 | 1.657 ± 1.247 | 0.315 | 1.017 | 0.378 |
| **Paracaedibacterales** | **0.373 ± 0.181** | **0.274 ± 0.186** | -1.087 | -2.642 | **0.018** |
| Pseudomonadales | 38.917 ± 15.460 | 29.332 ± 17.757 | -1.051 | -1.263 | 0.284 |
| Reyranellales | 0.334 ± 0.125 | 0.571 ± 0.816 | -0.128 | -0.271 | 0.786 |
| **Other** | **8.008 ± 3.718** | **6.102 ± 4.874** | -1.031 | -2.493 | **0.023** |
|  | | | | | |
| **C** Differential abundances at 17 days post-infection | | | | | |
| Order | %compositional mean ± standard deviation | | log2FoldChange | Wald Stat | padj |
|  | Control | NP-altered |  |  |  |
| Burkholderiales | 30.443 ± 6.077 | 39.199 ± 14.857 | -0.143 | -0.404 | 0.754 |
| **Caulobacterales** | **0.146 ± 0.133** | **0.986 ± 0.457** | -2.758 | -4.526 | **3.30e-05** |
| **Chitinophagales** | **0.108 ± 0.095** | **4.020 ± 2.261** | -5.139 | -5.792 | **7.65e-08** |
| Cytophagales | 10.619 ± 2.955 | 17.184 ± 7.529 | -0.417 | -1.011 | 0.429 |
| **Enterobacterales** | **6.436 ± 10.242** | **0.446 ± 0.298** | 4.347 | 4.098 | **1.53e-04** |
| Flavobacteriales | 8.643 ± 5.213 | 8.646 ± 4.114 | 0.479 | 0.626 | 0.649 |
| **Hyphomicrobiales** | **1.089 ± 0.429** | **2.379 ± 1.135** | -0.891 | -2.390 | **0.047** |
| Paracaedibacterales | 0.559 ± 0.252 | 0.388 ± 0.097 | 0.514 | 1.052 | 0.429 |
| Pseudomonadales | 28.853 ± 3.145 | 19.033 ± 28.274 | -0.315 | -0.314 | 0.754 |
| Reyranellales | 0.542 ± 0.385 | 0.932 ± 0.405 | -0.675 | -1.189 | 0.429 |
| Other | 12.562 ± 5.394 | 7.187 ± 2.954 | 0.863 | 1.729 | 0.184 |
|  | | | | | |
| **D** Differential abundances at 20 days post-infection | | | | | |
| Order | %compositional mean ± standard deviation | | log2FoldChange | Wald Stat | padj |
|  | Control | NP-altered |  |  |  |
| **Burkholderiales** | **11.507 ± 6.864** | **45.590 ± 10.164** | -1.323 | -3.052 | **0.008** |
| **Caulobacterales** | **0.188 ± 0.004** | **1.377 ± 0.563** | -1.869 | -2.530 | **0.025** |
| **Chitinophagales** | **0.135 ± 0.079** | **4.241 ± 4.95** | -4.152 | -3.828 | **0.001** |
| Cytophagales | 5.989 ± 0.733 | 17.420 ± 7.456 | -0.478 | -0.945 | 0.360 |
| Enterobacterales | 0.132 ± 0.067 | 0.724 ± 0.511 | -1.286 | -0.980 | 0.360 |
| Flavobacteriales | 7.449 ± 3.908 | 5.414 ± 5.355 | 1.417 | 1.511 | 0.205 |
| **Hyphomicrobiales** | **1.174 ± 0.900** | **4.830 ± 2.039** | -1.343 | -2.939 | **0.009** |
| Paracaedibacterales | 0.339 ± 0.131 | 0.464 ± 0.361 | 0.553 | 0.915 | 0.360 |
| **Pseudomonadales** | **61.428 ± 3.145** | **6.720 ± 3.476** | 4.137 | 3.363 | **0.004** |
| Reyranellales | 1.334 ± 1.387 | 3.541 ± 1.280 | -0.897 | -1.302 | 0.265 |
| Other | 10.327 ± 1.288 | 9.678 ± 5.634 | 1.037 | 1.696 | 0.164 |

**References (Table S5)**

1. Chen WM, Chen WT, Young CC, Sheu SY. Rheinheimera riviphila sp. nov., isolated from a freshwater stream. *Arch. Microbiol.* 2019;**201**:919–926. https://doi.org/10.1007/S00203-019-01657-5

2. Chiellini C, Pasqualetti C, Lanzoni O, Fagorzi C, Bazzocchi C, Fani R, et al. Harmful effect of Rheinheimera sp. Eprs3 (Gammaproteobacteria) against the protist Euplotes aediculatus (Ciliophora, Spirotrichea): Insights into the ecological role of antimicrobial compounds from environmental bacterial strains. *Front. Microbiol.* 2019;**10**. https://doi.org/10.3389/fmicb.2019.00510

3. Fu J, Zhong C, Zhang P, Gao Q, Zong G, Zhou Y, et al. A Novel Mobile Element ICERspD18B in Rheinheimera sp. D18 contributes to antibiotic and arsenic resistance. *Front. Microbiol.* 2020;**11**:616364. https://doi.org/10.3389/fmicb.2020.616364

4. Li T, Zhuo Y, Jin CZ, Wu X, Ko SR, Jin FJ, et al. Genomic insights into a novel species Rhodoferax aquaticus sp. nov., isolated from freshwater. *Int J Syst Evol Microbiol* 2020;70:4653–4660. https://doi.org/10.1099/ijsem.0.004325

5. Baker JM, Riester CJ, Skinner BM, Newell AW, Swingley WD, Madigan MT, et al. Genome sequence of Rhodoferax antarcticus ANT.BRT; a psychrophilic purple nonsulfur bacterium from an Antarctic microbial mat. *Microorganisms* 2017;**5**:8. https://doi.org/10.3390/microorganisms5010008

6. Jin CZ, Zhuo Y, Wu, X, Ko SR, Li T, Jin FJ, et al. Genomic and metabolic insights into denitrification, sulfur oxidation, and multidrug efflux pump mechanisms in the bacterium Rhodoferax sediminis sp. nov. *Microorganisms* 2020;**8**:262. https://doi.org/10.3390/microorganisms8020262

7. Nakai R, Fujisawa T, Nakamura Y, Baba T, Nishijima M, Karray F, et al. Genome sequence and overview of Oligoflexus tunisiensis Shr3T in the eighth class Oligoflexia of the phylum Proteobacteria. *Stand Genomic Sci* 2016;**11**:90. https://doi.org/10.1186/s40793-016-0210-6

8. Gu HY, Hao SY, Sun ZB, Xu JL, Ren Q, Pan HX. Bosea beijingensis sp. nov., Telluria beijingensis sp. nov. and Agrococcus beijingensis sp. nov., isolated from baijiu mash. *Int J Syst Evol Microbiol* 2024;**74**:006304. https://doi.org/10.1099/ijsem.0.006304

9. Walczak AB, Yee N, Young LY. Draft genome sequence of Bosea sp. WAO an arsenite and sulfide oxidizer isolated from a pyrite rock outcrop in New Jersey. *Stand Genomic Sci* 2018;**13**:1–12. https://doi.org/10.1186/s40793-018-0312-4

10. Wu Y, Xiang L, Wang H, Ma L, Qiu X, Liu D, et al. Transcriptome analysis of an arsenite-/antimonite-oxidizer, Bosea sp. AS-1 reveals the importance of the type 4 secretion system in antimony resistance. *Sci. Total Environ.* 2022;**826**. https://doi.org/10.1016/j.scitotenv.2022.154168

11. Sazanova AL, Safronova, VI, Kuznetsova IG, Karlov DS, Belimov AA, Andronov EE, et al. Bosea caraganae sp. nov. a new species of slow-growing bacteria isolated from root nodules of the relict species Caragana jubata (Pall.) poir. originating from Mongolia. *Int J Syst Evol Microbiol.* 2019;**69**:2687–2695. https://doi.org/10.1099/ijsem.0.003509

12. Sun LN, Yang ED, Hou XT, Wei JC, Yuan ZX, Wang WY. Caulobacter Rhizosphaerae sp. nov., a stalked bacterium isolated from rhizosphere soil. *Int J Syst Evol Microbiol* 2017;**67**:1771–1776. https://doi.org/10.1099/ijsem.0.001860

13. Yang Y, Jin CZ, Jin FJ, Li T, Lee JM, Kim CJ, et al. Caulobacter soli sp. nov., isolated from soil sampled at Jiri Mountain, Republic of Korea. *Int J Syst Evol Microbiol* 2020;**70**:4158–4164. https://doi.org/10.1099/ijsem.0.004264

14. Poindexter JS. Biological properties and classification of the Caulobacter group. *Bacteriol Rev* 1964;**28**:231–295. https://doi.org/10.1128/br.28.3.231-295.1964

15. Fiebig A. Role of Caulobacter cell surface structures in colonization of the air-liquid interface. *J Bacteriol* 2019;**201**. https://doi.org/10.1128/jb.00064-19

16. Zhang M, Zhang Y, Fan Y, Yao Q, Zhu H. Gemmobacter denitrificans sp. nov., a denitrifying bacterium, isolated from pond water for Litopenaeus vannamei. Int *J Syst Evol Microbiol.* 2024;**74**:006430. https://doi.org/10.1099/ijsem.0.006430

17. Sheu SY, Shiau YW, Wei YT, Chen WM. Gemmobacter lanyuensis sp. nov., isolated from a freshwater spring. *Int J Syst Evol Microbiol* 2013;**63**:4039–4045. https://doi.org/10.1099/ijs.0.052399-0

18. Qu JH, Ma WW, Zhou J, Wang XF, Lu WL, Qu LB, et al. Gemmobacter caeruleus sp. nov., a novel species originating from lake sediment. *Int J Syst Evol Microbiol* 2020;**70**:1987–1992. https://doi.org/10.1099/ijsem.0.004007

19. Rothe B, Fischer A, Hirsch P, Stackebrandt E. The phylogenetic position of the budding bacteria Blastobacter aggregatus and Gemmobacter aquatilis gen., nov. sp. nov. *Arch Microbiol* 1987;**147**:92–99. https://doi.org/10.1007/BF00492911

20. Chen WM, Cho NT, Huang WC, Young CC, Sheu SY. Description of Gemmobacter fontiphilus sp. nov., Isolated from a Freshwater Spring, Reclassification of Catellibacterium nectariphilum as Gemmobacter nectariphilus comb. nov., Catellibacterium changlense as Gemmobacter changlensis comb. nov., Catellibacterium aquatile as Gemmobacter aquaticus nom. nov., Catellibacterium caeni as Gemmobacter caeni comb. nov., Catellibacterium nanjingense as Gemmobacter nanjingensis comb. nov., and emended description of the genus Gemmobacter and of Gemmobacter aquatilis*.* *Int J Syst Evol Microbiol* 2013;**63**:470–478. https://doi.org/10.1099/ijs.0.042051-0

21. Jin L, Jin CZ, Lee HG, Lee CS. Genomic insights into denitrifying methane-oxidizing bacteria Gemmobacter fulva sp. nov., isolated from an Anabaena culture. *Microorganisms* 2021;**9**:2423. https://doi.org/10.3390/microorganisms9122423

22. Kröber E, Cunningham MR, Peixoto J, Spurgin L, Wischer D, Kruger R, et al. Comparative genomics analyses indicate differential methylated amine utilization trait within members of the genus Gemmobacter. *Environ Microbiol Rep* 2021;**13**:195–208. https://doi.org/10.1111/1758-2229.12927

23. Baik KS, Choe HN, Park SC, Hwang YM, Kim EM, Park C, et al. Sphingopyxis rigui sp. nov. and Sphingopyxis wooponensis sp. nov., isolated from wetland freshwater, and emended description of the genus Sphingopyxis. *Int J Syst Evol Microbiol* 2013;**63**:1297–1303. https://doi.org/10.1099/ijs.0.044057-0

24. Glaeser SP, Kämpfer P. The Family Sphingomonadaceae. In: Rosenberg E, DeLong EF, Lory S, Stackebrandt E, Thompson F (eds). *The Prokaryotes*. Berlin Heidelberg: Springer 2014. https://doi.org/10.1007/978-3-642-30197-1_302

25. Jeong HI, Jin HM, Jeon CO. Complete genome sequence of Sphingorhabdus sp. M41, a versatile hydrocarbon degrader, isolated from crude oil-contaminated costal sediment. *J Biotechnol* 2016;**227**:41–42. https://doi.org/10.1016/j.jbiotec.2016.04.016

26. Woo JH, Kim HS, Park NH, Suk HY. Isolation of a novel strain, Sphingorhabdus sp. YGSMI21 and characterization of its enantioselective epoxide hydrolase activity. *J. Microbiol.* 2021;**59**:675–680. https://doi.org/10.1007/s12275-021-1023-x

27. Hirose S, Tank M, Hara E, Tamaki H, Mori K, Takaichi S, et al*.* Aquabacterium pictum sp. nov., the first aerobic bacteriochlorophyll a-containing fresh water bacterium in the genus Aquabacterium of the class Betaproteobacteria. *Int J Syst Evol Microbiol* 2020;**70**:596–603. https://doi.org/10.1099/ijsem.0.003798

28. Hu Y, Liu T, Chen N, Feng C. Changes in microbial community diversity, composition, and functions upon nitrate and Cr(VI) contaminated groundwater. *Chemosphere* 2022;**288**:132476. https://doi.org/10.1016/j.chemosphere.2021.132476

29. Yoon JH, Kang SJ, Ryu SH, Jeon OK, Oh TK.Hydrogenophaga bisanensis sp. nov., isolated from wastewater of a textile dye works. *Int J Syst Evol Microbiol* 2008;**58**:393–397. https://doi.org/10.1099/ijs.0.65271-0

30. Kimura ZI, Okabe S. Acetate oxidation by syntrophic association between Geobacter sulfurreducens and a hydrogen-utilizing exoelectrogen. *ISME J.* 2013;**7**:1472–1482. https://doi.org/10.1038/ismej.2013.40

31. Belykh MP, Petrov SV, Chikin AJ, Belkova NL. Genetic diversity of bacteria adapted to cyanide-bearing compounds in the technogenic ecosystems as detected by 16S rDNA sequences. *Contemp Probl Ecol* 2016;**9**:563–573. https://doi.org/10.1134/S1995425516050012

32. Jewell TNM, Karaoz U, Bill M, Chakraborty R, Brodie EL, Williams KH, et al. Metatranscriptomic analysis reveals unexpectedly diverse microbial metabolism in a biogeochemical hot spot in an alluvial aquifer. *Front Microbiol* 2017;**8**:235809. https://doi.org/10.3389/fmicb.2017.00040

33. Willems A. The Family Comamonadaceae. In: Rosenberg E, DeLong EF, Lory S, Stackebrandt E, Thompson F (eds). *The Prokaryotes*. Berlin, Heidelberg: Springer 2014. https://doi.org/10.1007/978-3-642-30197-1_238

34. McIlroy SJ, Nielsen PH. The Family Saprospiraceae.  In: Rosenberg E, DeLong EF, Lory S, Stackebrandt E, Thompson F (eds). *The Prokaryotes*. Berlin, Heidelberg: Springer 2014. https://doi.org/10.1007/978-3-642-38954-2_138

35. Xia Y, Kong Y, Thomsen TR, Nielsen PH. Identification and ecophysiological characterization of epiphytic protein-hydrolyzing Saprospiraceae (‘Candidatus epiflobacter’ spp.) in activated sludge. *Appl Environ Microbiol* 2008;**74**:2229–2238. https://doi.org/10.1128/aem.02502-07

36. Midha S, Rigden DJ, Siozios S, Hurst GDD, Jackson AP. Bodo saltans (Kinetoplastida) is dependent on a novel Paracaedibacter-like endosymbiont that possesses multiple putative toxin-antitoxin systems. *ISME J.* 2021;**15**:1680–1694. https://doi.org/10.1038/S41396-020-00879-6

37. Lanzoni O, Szokoli F, Schrallhammer M, Sabaneyeva E, Krenek S, Doak TG, et al. “Candidatus Intestinibacterium parameciiphilum”—member of the “Candidatus Paracaedibacteraceae” family (Alphaproteobacteria, Holosporales) inhabiting the ciliated protist Paramecium. *Int. Microbiol.* 2024;**27**:659–671. https://doi.org/10.1007/s10123-023-00414-5

38. Pouder E, Vince E, Jacquot K, Traoré M, Grosche A, Ludwig M, et al. Phenylobacterium ferrooxidans sp. nov., isolated from a sub-surface geothermal aquifer in Iceland. *Syst Appl Microbiol* 2025;**48**:126578. https://doi.org/10.1016/j.syapm.2024.126578

39. Kim JW, Jo JH, Im WT. Dechloromonas hankyongensis sp. nov., isolated from wetland. *Korean J. Microbiol.* 2023;**59**:8–15. https://doi.org/10.7845/kjm.2023.3015

40. Zhang S, Amanze C, Sun C, Zou K, Fu S, Deng Y, et al. Evolutionary, genomic, and biogeographic characterization of two novel xenobiotics-degrading strains affiliated with Dechloromonas. *Heliyon* 2021;**7**:e07181. https://doi.org/10.1016/j.heliyon.2021.e07181

41. Abraham WR, Strömpl C, Meyer H, Lindholst S, Moore ERB, Christ R, et al. Phylogeny and polyphasic taxonomy of Caulobacter species. Proposal of Maricaulis gen. nov. with Maricaulis maris (Poindexter) comb. nov. as the type species, and emended description of the genera Brevundimonas and Caulobacter. *Int J Syst Bacteriol* 1999;**49**:1053–1073. https://doi.org/10.1099/00207713-49-3-1053

42. Maltman C, Messner K, Kyndt JA, Yurkov V. Brevundimonas aurifodinae, sp. nov., an aerobic anoxygenic phototroph resistant to metalloid oxyanions isolated from gold mine tailings. *Microorganisms* 2024;**12**:2167. https://doi.org/10.3390/microorganisms12112167

43. Tsubouchi T, Koyama S, Mori K, Shimane Y, Usui K, Tokuda M, et al. Brevundimonas denitrificans sp. nov., a denitrifying bacterium isolated from deep subseafloor sediment. *Int J Syst Evol Microbiol* 2014;**64**:3709–3716. https://doi.org/10.1099/ijs.0.067199-0

44. Macedo AJ, Timmis KN, Abraham WR. Widespread capacity to metabolize polychlorinated biphenyls by diverse microbial communities in soils with no significant exposure to PCB contamination. *Environ Microbiol* 2007;**9**:1890–1897. https://doi.org/10.1111/j.1462-2920.2007.01305.x

45. Leandro T, França L, Nobre MF, Schumann P, Rosselló-Móra R, da Costa MS. Nevskia aquatilis sp. nov. and Nevskia persephonica sp. nov., isolated from a mineral water aquifer and the emended description of the genus Nevskia. *Syst Appl Microbiol* 2012;**35**:297–301. https://doi.org/10.1016/j.syapm.2012.05.001

46. Jiang Y, Jiang L, Peng Y, Kim KH, Shin HH, Kim YM, et al. Flagellatimonas centrodinii gen. nov., sp. nov., a novel member of the family Nevskiaceae isolated from toxin-producing dinoflagellate Centrodinium punctatum. *Int J Syst Evol Microbiol* 2021;**71**:005084. https://doi.org/10.1099/ijsem.0.005084

47. Fahrbach M, Kuever J, Remesch M, Huber BE, Kämpfer P, Dott W, et al. Steroidobacter denitrificans gen. nov., sp. nov., a steroidal hormone-degrading Gammaproteobacterium. *Int J Syst Evol Microbiol* 2008;**58**:2215–2223. https://doi.org/10.1099/ijs.0.65342-0

48. Banerjee S, Táncsics A, Tóth E, Révész F, Bóka K, Kriszt B. Hydrogenophaga aromaticivorans sp. Nov., isolated from a para-xylene-degrading enrichment culture, capable of degrading benzene, meta-and para-xylene. *Int J Syst Evol Microbiol* 2021;**71**:004743 https://doi.org/10.1099/ijsem.0.004743

49. Fahy A, Ball AS, Lethbridge G, Timmis KN, McGenity TJ. Isolation of alkali‐tolerant benzene‐degrading bacteria from a contaminated aquifer. *Lett Appl Microbiol* 2008;**47**:60–66. https://doi.org/10.1111/j.1472-765x.2008.02386.x

50. Glaeser SP, Bolte K, Martin K, Busse HJ, Grossart HP, Kämpfer P, et al. Novosphingobium fuchskuhlense sp. nov., isolated from the north-east basin of Lake Grosse Fuchskuhle. *Int J Syst Evol Microbiol* 2013;**63**:586–592. https://doi.org/10.1099/ijs.0.043083-0

51. Hu W, Li Z, Ou H, Wang X, Wang Q, Tao Z, et al. Novosphingobium album sp. nov., Novosphingobium organovorum sp. nov. and Novosphingobium mangrovi sp. nov. with the organophosphorus pesticides degrading ability isolated from mangrove sediments. *Int J Syst Evol Microbiol* 2023;**73**:005843. https://doi.org/10.1099/ijsem.0.005843

52. Fujii K, Satomi M, Morita N, Motomura T, Tanaka T, Kikuchi S. Novosphingobium tardaugens sp. nov., an oestradiol-degrading bacterium isolated from activated sludge of a sewage treatment plant in Tokyo. *Int J Syst Evol Microbiol* 2003;**53**:47–52. https://doi.org/10.1099/ijs.0.02301-0

53. Liu ZP, Wang BJ, Liu YH, Liu SJ. Novosphingobium taihuense sp. nov., a novel aromatic-compound-degrading bacterium isolated from Taihu Lake, China. *Int J Syst Evol Microbiol* 2005;**55**:1229–1232. https://doi.org/10.1099/ijs.0.63468-0

54. Sohn JH, Kwon KK, Kang JH, Jung HB, Kim SJ. Novosphingobium pentaromativorans sp. nov., a high-molecular-mass polycyclic aromatic hydrocarbon-degrading bacterium isolated from estuarine sediment. *Int J Syst Evol Microbiol* 2004;**54**:1483–1487. https://doi.org/10.1099/ijs.0.02945-0

55. Cui Y, Park CY, Kim SY, Wong SK, Kim AS, Oh HM, et al. Aquariibacter albus gen. Nov., sp. nov., a new member of the order burkholderiales, isolated from a freshwater aquarium. *Int J Syst Evol Microbiol* 2021;**71**:005022. https://doi.org/10.1099/ijsem.0.005022

56. Stackebrandt E, Verbarg S, Frühling A, Busse HJ, Tindall BJ. Dissection of the genus Methylibium: Reclassification of Methylibium fulvum as Rhizobacter fulvus comb. nov., Methylibium aquaticum as Piscinibacter aquaticus gen. nov., comb. nov. and Methylibium subsaxonicum as Rivibacter subsaxonicus gen. nov., comb. nov. and emended descriptions of the genera Rhizobacter and Methylibium. *Int J Syst Evol Microbiol* 2009;**59**:2552–2560. https://doi.org/10.1099/ijs.0.008383-0

57. Manakul P, Peerakietkhajorn S, Matsuura T, Kato Y, Watanabe H. Effects of symbiotic bacteria on chemical sensitivity of Daphnia magna. *Mar Environ Res* 2017;**128**:70–75. https://doi.org/10.1016/j.marenvres.2017.03.001

58. Peerakietkhajorn S, Kato Y, Kasalický V, Matsuura T, Watanabe H. Betaproteobacteria Limnohabitans strains increase fecundity in the crustacean Daphnia magna: symbiotic relationship between major bacterioplankton and zooplankton in freshwater ecosystem. *Environ Microbiol* 2016;**18**:2366–2374. https://doi.org/10.1111/1462-2920.12919

59. Peerakietkhajorn S, Tsukada K, Kato Y, Matsuura T, Watanabe H. Symbiotic bacteria contribute to increasing the population size of a freshwater crustacean, Daphnia magna. *Environ Microbiol Rep* 2015;**7**:364–372. https://doi.org/10.1111/1758-2229.12260

60. Delgado MJ, Casella S, Bedmar EJ. Denitrification in Rhizobia-Legume Symbiosis. *Biology of the Nitrogen Cycle* 2006;**83**,IV-91,IV. https://doi.org/10.1016/B978-044452857-5.50007-2

61. Sánchez C, Mercante V, Babuin MF, Lepek VC. Dual effect of Mesorhizobium loti T3SS functionality on the symbiotic process. *FEMS Microbiol Lett* 2012;**330**:148–156. https://doi.org/10.1111/j.1574-6968.2012.02545.x

62. Riquelme C, Hathaway JJM, Dapkevicius M, Miller AZ, Kooser A, Northup DE, et al. Actinobacterial diversity in volcanic caves and associated geomicrobiological interactions. *Front Microbiol* 2015;**6**:162211. https://doi.org/10.3389/fmicb.2015.01342

63. Thirumurugan D, Vijayakumar R, Vadivalagan C, Karthika P, Khan MKA. Isolation, structure elucidation and antibacterial activity of methyl-4,8-dimethylundecanate from the marine actinobacterium Streptomyces albogriseolus ECR64. *Microb Pathog* 2018;**121**:166–172. https://doi.org/10.1016/j.micpath.2018.05.025
