# Supplementary figures and images for: Nanoplastic-induced microbiome shifts reduce Daphnia fitness and increase parasite reproduction
Source: ISME Commun. 2026 Apr 20;6(1):ycag109. doi: 10.1093/ismeco/ycag109 (PMC13196593; doi:10.1093/ismeco/ycag109)

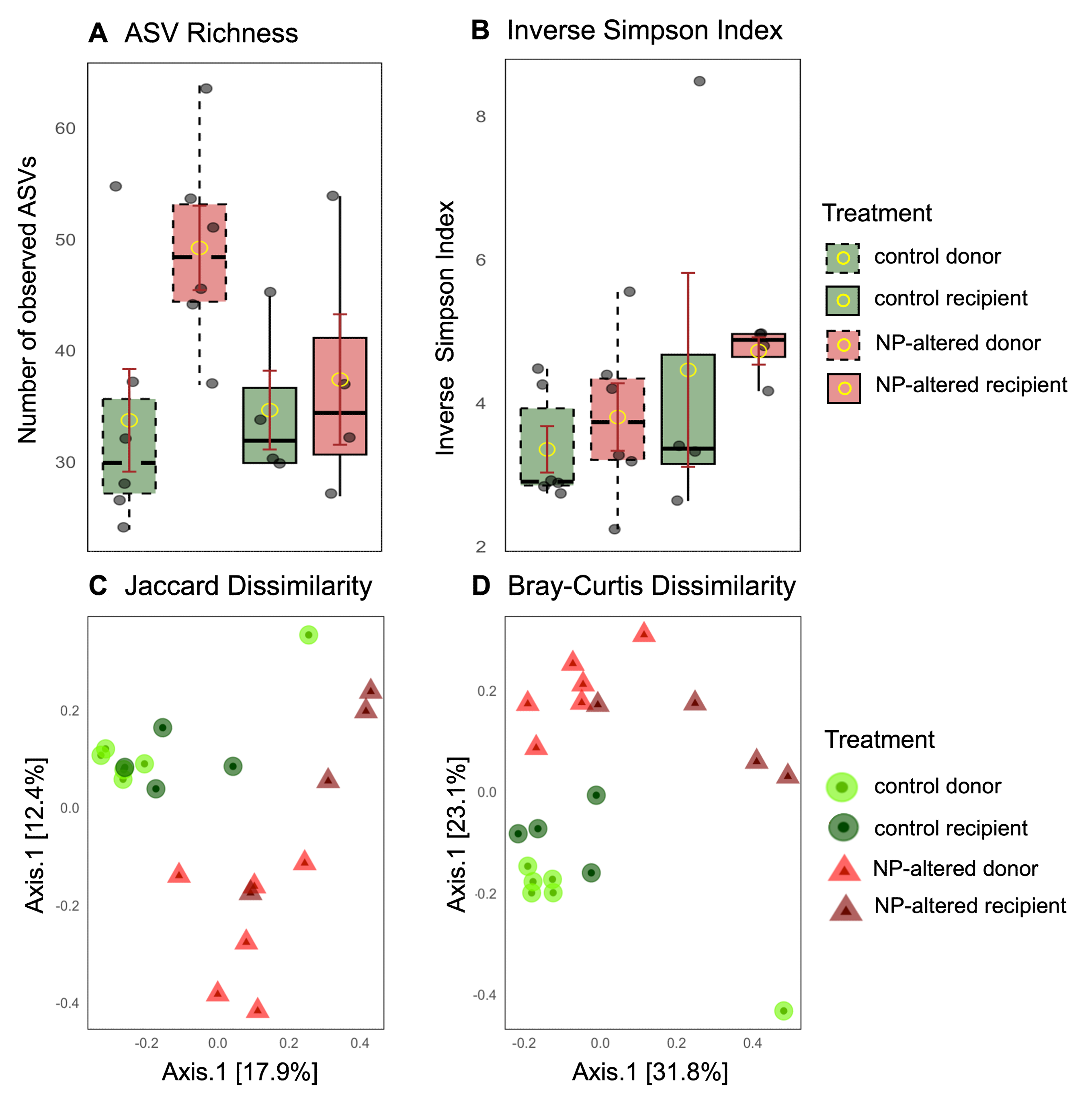

Supplement: Supplementary_Material_ycag109 [file supplementary_material_ycag109.zip › Figure S3.png]

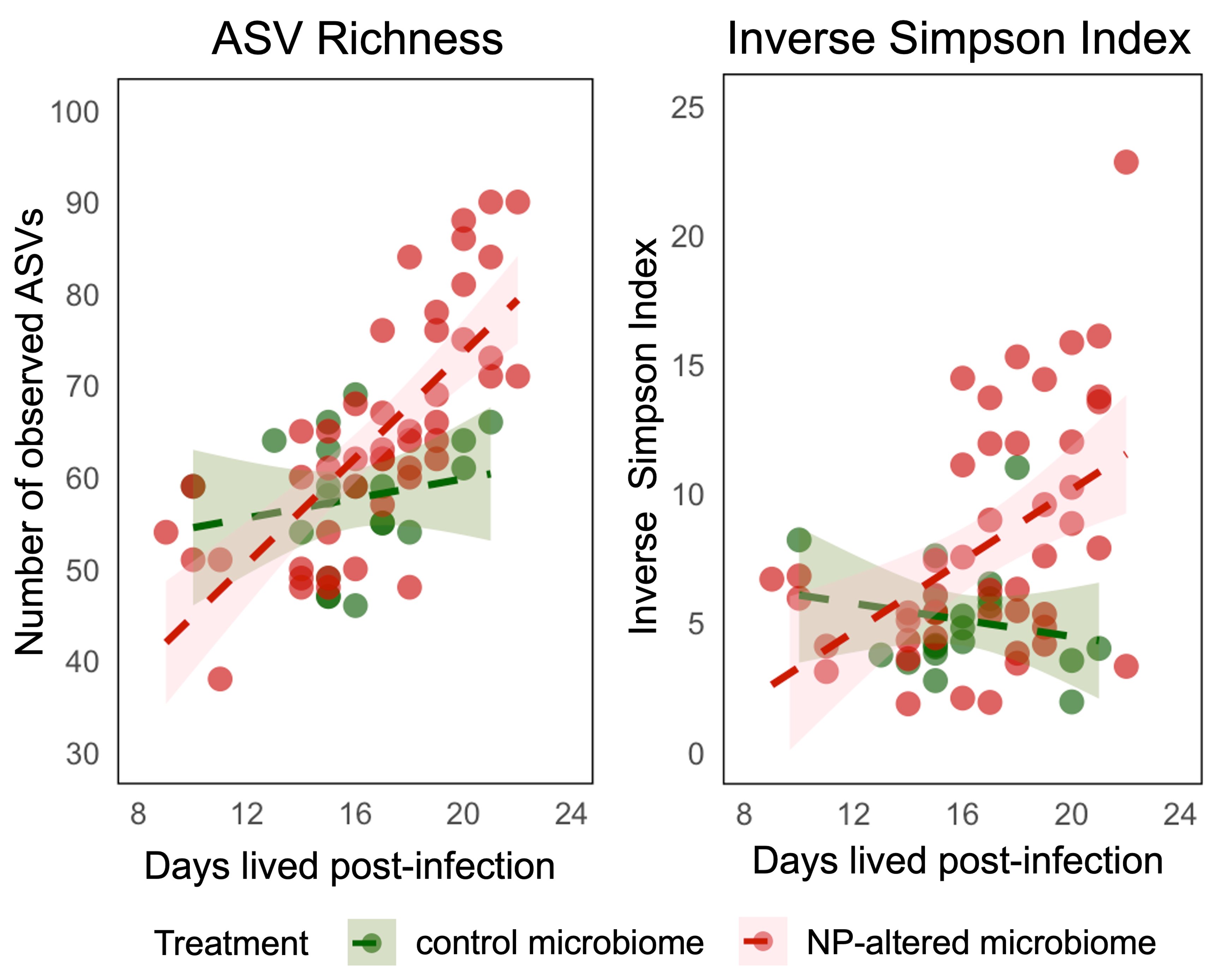

Supplement: Supplementary_Material_ycag109 [file supplementary_material_ycag109.zip › Figure S4.png]

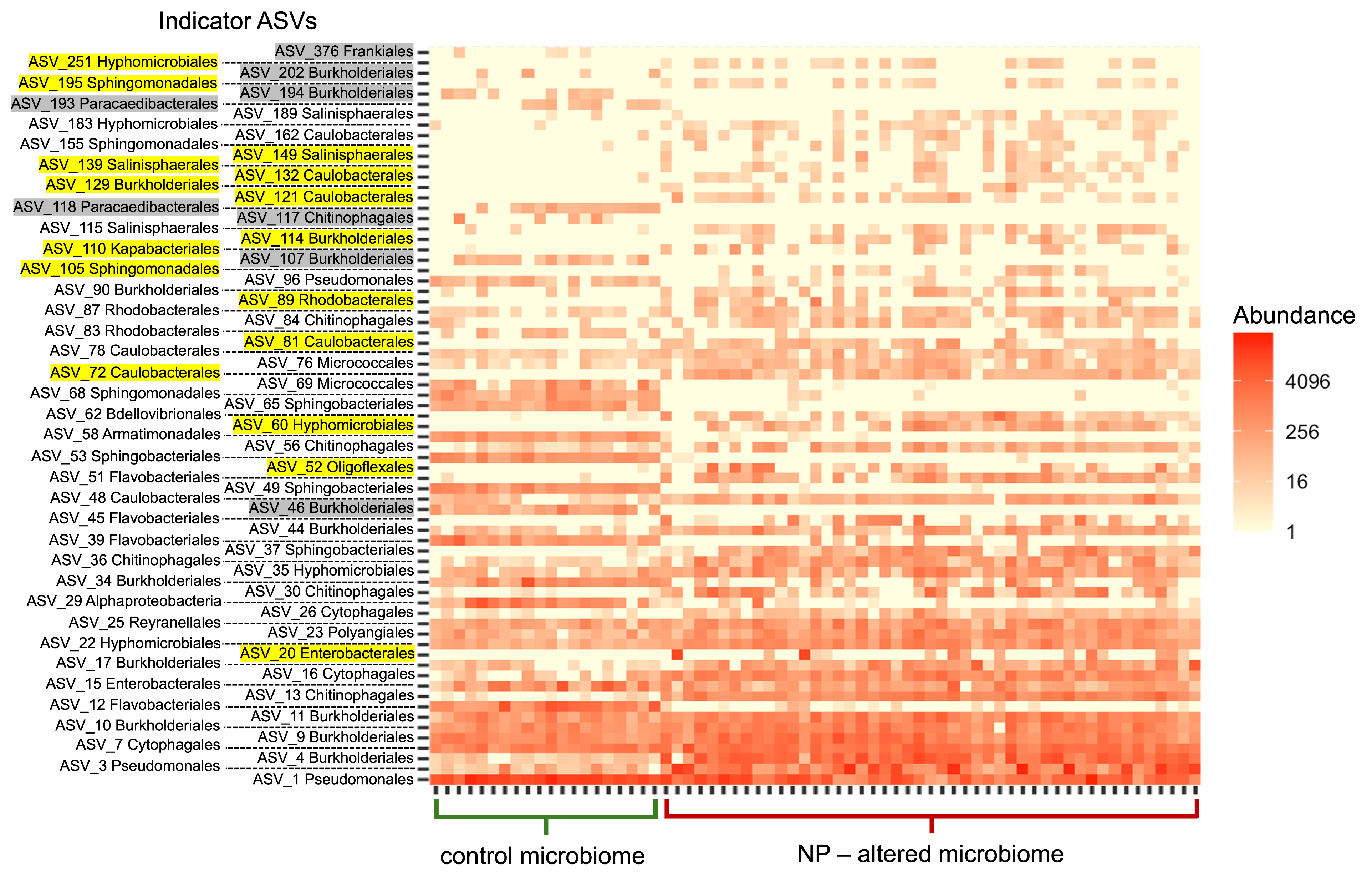

Supplement: Supplementary_Material_ycag109 [file supplementary_material_ycag109.zip › Figure S5.png]

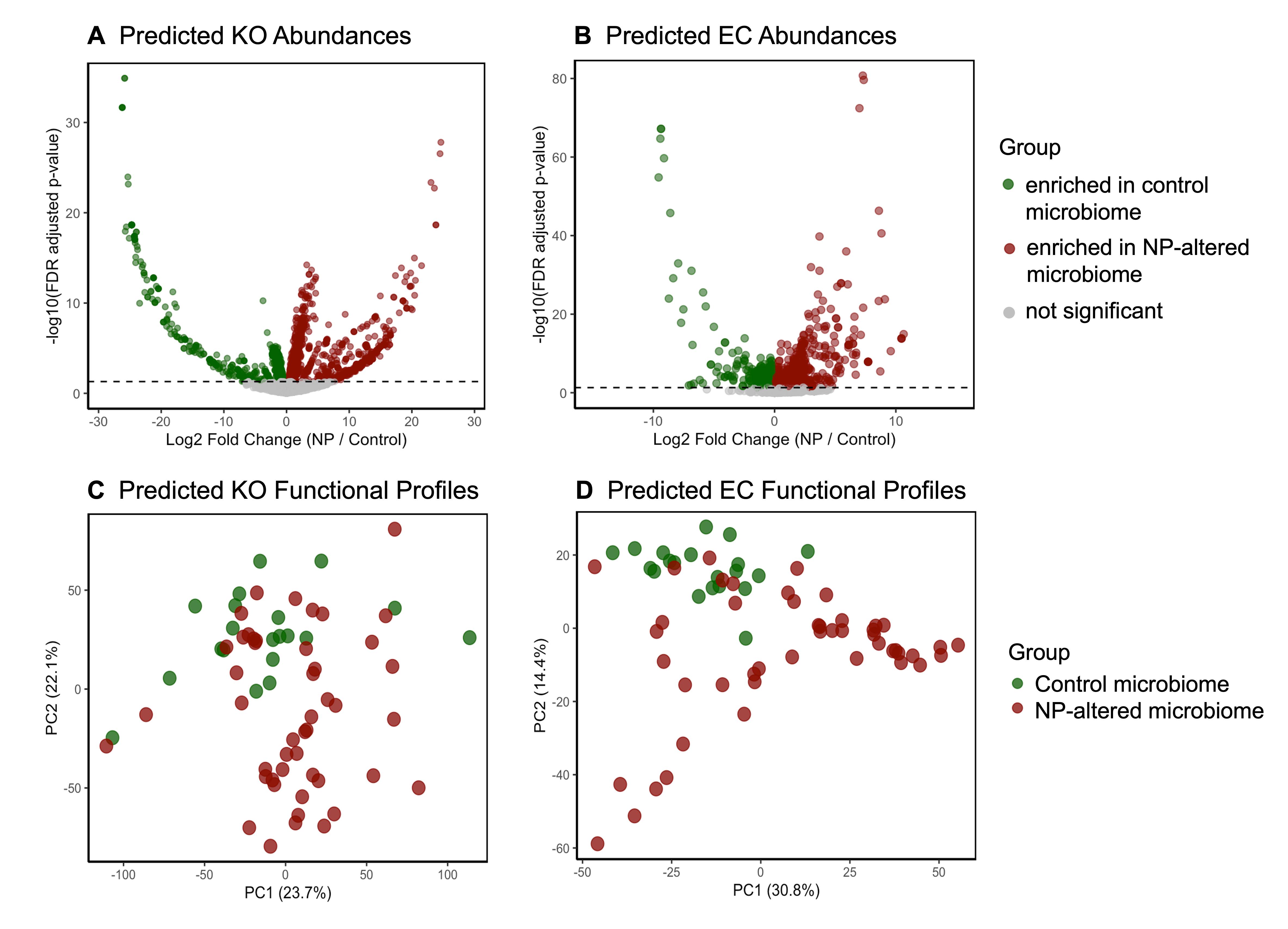

Supplement: Supplementary_Material_ycag109 [file supplementary_material_ycag109.zip › Figure S6.png]

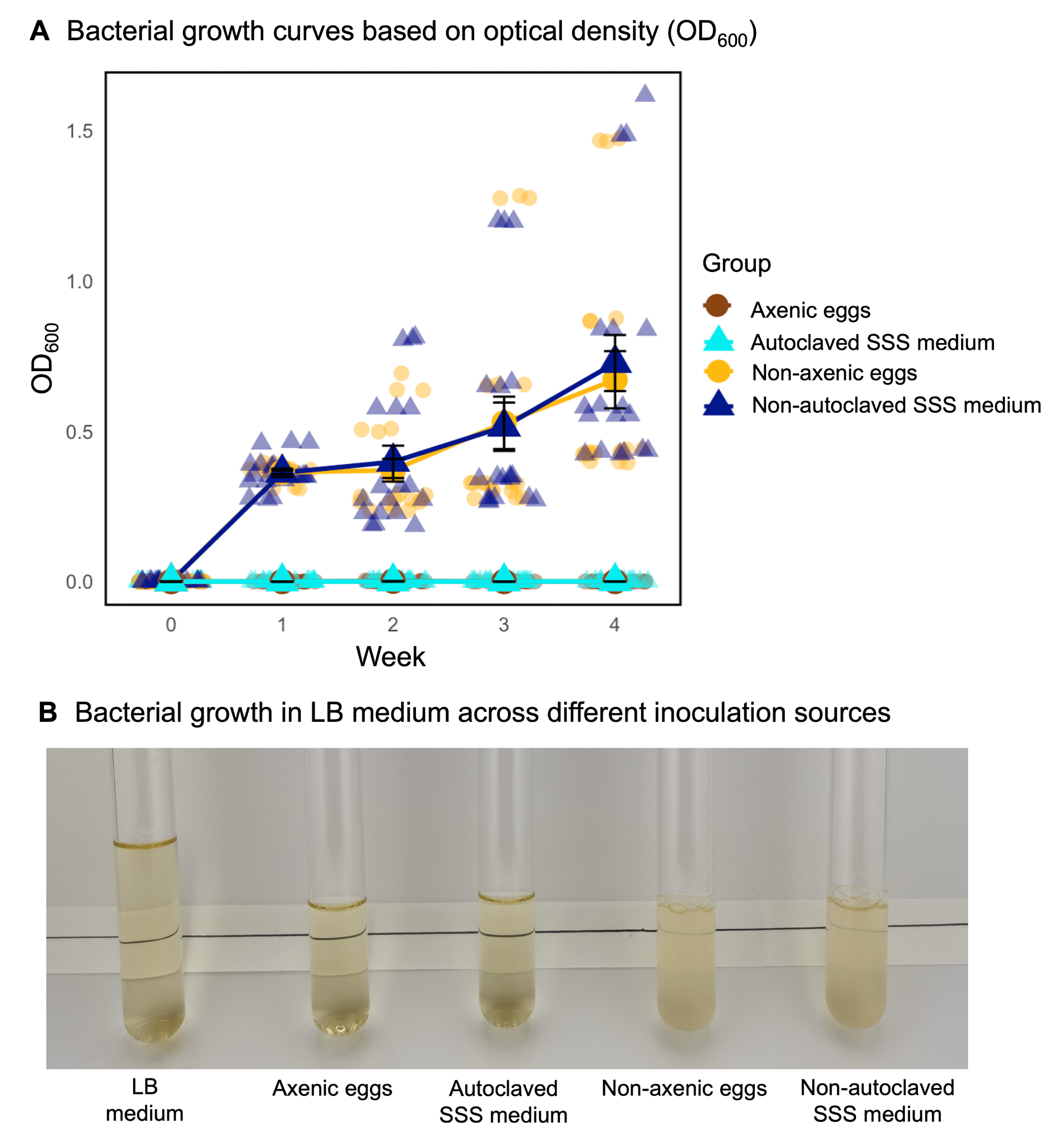

Supplement: Supplementary_Material_ycag109 [file supplementary_material_ycag109.zip › Figure S1.png]

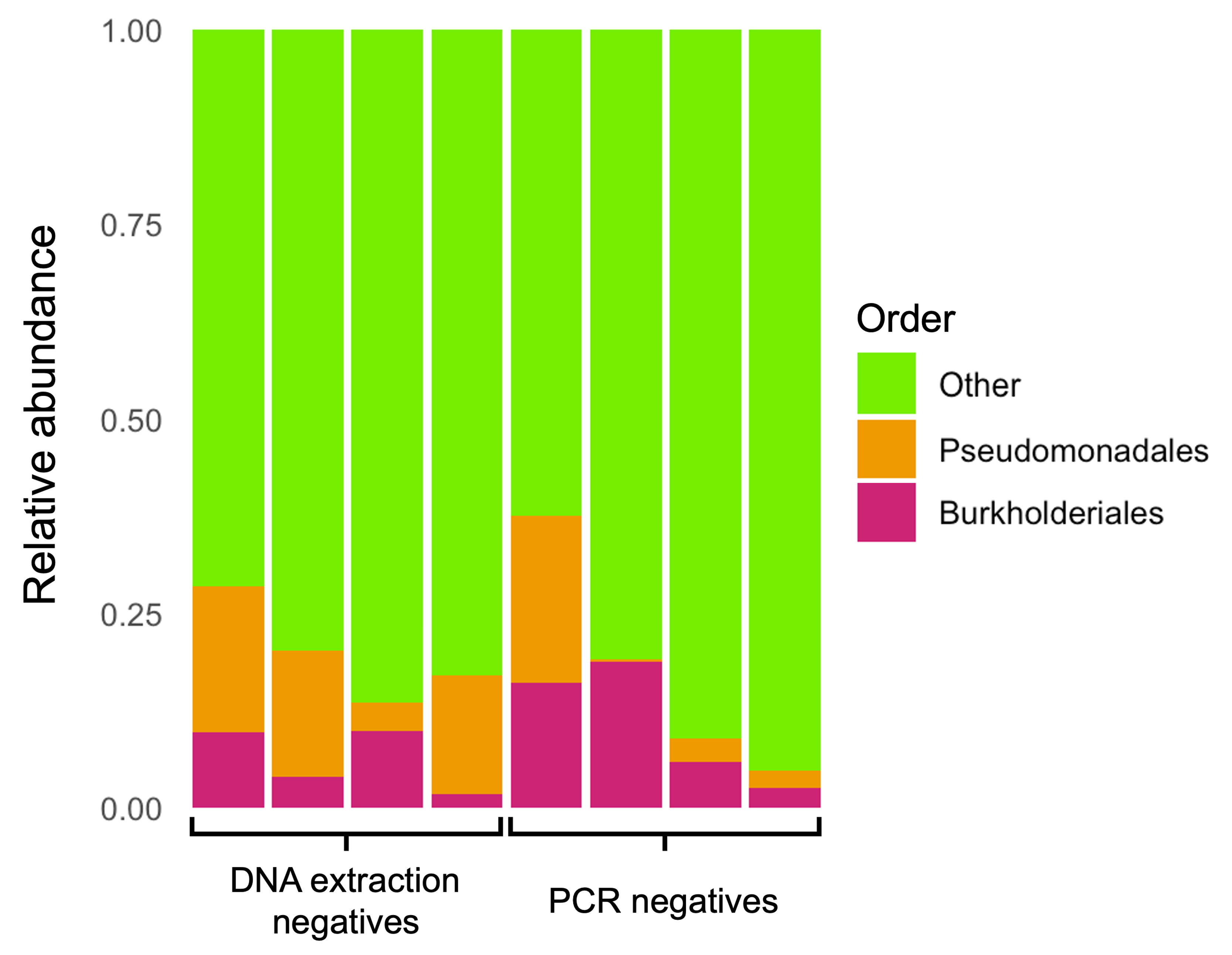

Supplement: Supplementary_Material_ycag109 [file supplementary_material_ycag109.zip › Figure S2.png]
